# Hofmeister-Driven Ion Pairing in Monovalent Salts Directs Fibrinogen Nanofiber Assembly during Drying

**DOI:** 10.1021/acs.biomac.5c01056

**Published:** 2025-09-05

**Authors:** Stephani Stamboroski, Aparna Sai Malisetty, Kwasi Boateng, Jana Lierath, Jonas Aniol, Peter Schiffels, Paul-Ludwig Michael Noeske, Lucio Colombi Ciacchi, Susan Köppen, Dorothea Brüggemann

**Affiliations:** 1 28456 Fraunhofer Institute for Manufacturing Technology and Advanced Materials IFAM, Wiener Strasse 12, Bremen 28359, Germany; 2 Institute for Biophysics, 9168 University of Bremen, Otto-Hahn-Allee 1, Bremen 28359, Germany; 3 Hybrid Materials Interfaces Group, Faculty of Production Engineering and Bremen Center for Computational Materials Science, 9168 University of Bremen, Bremen 28359, Germany; 4 University of Applied Sciences Bremerhaven, An der Karlstadt 8, Bremerhaven 27568, Germany; 5 City University of Applied Sciences, Neustadtswall 30, Bremen 28199, Germany; 6 MAPEX Center for Materials and Processes, 9168 University of Bremen, Bremen 28359, Germany

## Abstract

Fibrinogen nanofiber scaffolds hold promise for tissue engineering and wound healing due to their similarity to fibrin clots. We studied how alkaline salts (Na^+^, K^+^) influence fibrinogen precipitation during drying of highly saline dispersions. In situ roughness (Aq) monitoring revealed coprecipitation of salts and fibrinogen. SEM and Aq mapping showed morphologies from smooth (KCl) and faintly fibrous (NaCl) to highly rough and finely fibrous (Na-PO_4_, K-PO_4_). FTIR indicated that secondary structure changes are not always linked to fiber formation. XPS showed a stronger Na^+^ uptake, especially with fiber-forming salts. With Na^+^ and oxygen-containing polyvalent anions, kosmotropic SO_4_^2^^–^ induced fibers, while chaotropic oxalate yielded smooth films. Mg^2^^+^ or K^+^ with SO_4_^2^^–^ did not form any fibers. Molecular dynamics simulations suggest ion-specific binding at the fibrinogen/water interface. We propose a two-dimensional Hofmeister series for tailoring fibrillogenesis via kosmotropic anion–cation pairs, concluding that fiber assembly is salt-driven and governed by cooperative kosmotropic effects.

## Graphical abstract

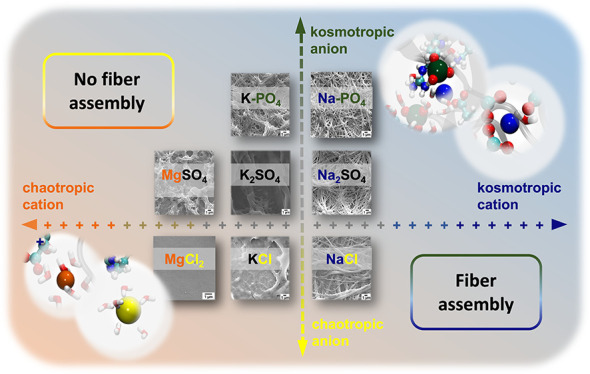

## 1 Introduction

The blood glycoprotein fibrinogen plays a crucial role in blood coagulation and wound healing, contributing to the formation of nanofibrous blood clots,^1,2^ which makes it predestined to prepare fibrous scaffolds for regenerative medicine. Fibrinogen has a molecular weight of 340 kDa and consists of a central globular E region and two identical D regions, both containing three pairs of polypeptide chains (Aα, Bβ, and γ) that are covalently linked by five disulfide bonds in the E region. Fibrinopeptides (FpA and FpB) are located at the ends of the Aα and Bβ chains within the E region. Flexible αC domains with disordered structure extend from the distal coiled-coil region of each Aα chain to the central region, interacting with each other and the central region of the molecule.^2–4^

Fibrinogen is present in blood plasma at concentrations of 1.5 to 4 mg mL^–1^,^3,5^ surrounded by numerous ions.^6,7^ In healthy humans, the major cations in the blood plasma are Na^+^ (142 mM), K^+^ (5 mM), Mg^2+^ (1.5 mM), and Ca^2+^ (2.5 mM), together with the anions Cl^–^ (103 mM), HCO_3_^–^ (27 mM), HPO_4_^2–^ (1 mM), and SO_4_^2–^ (0.5 mM).^7,8^ In addition to assisting blood coagulation, these ions are important for many biochemical functions, such as buffering the pH of blood and maintaining the osmotic pressure or muscle activity.^3,6,9^ Mediated by Ca^2+^ ions, the enzyme thrombin cleaves FpA and FpB from fibrinogen, leading to its ordered assembly into fibrin, the fundamental building block of blood clots.^3^ *In vivo,* this polymerization initiates the formation of an insoluble nanofiber network that serves as a scaffold for platelet adhesion, preventing blood loss after injury. Numerous *in vitro* strategies have been developed to reproduce this 3D nanostructure that serves as a provisional extracellular matrix during tissue repair.

Fibrinogen molecules themselves, for instance, can assemble into nanofibrous networks without the presence of thrombin under various *in vitro* conditions that we previously grouped into three categories: substrate interaction, denaturing buffers, and nondenaturing buffers.^1^ The diameters of fibrinogen fibers prepared *in vitro* vary widely, ranging from 3 to 300 nm, depending on the technique used to induce fiber formation.^1^ Electrospinning, the most common technique, yields fibrinogen nanofibers with good biocompatibility.^10–13^ However, high fibrinogen amounts^14,15^ are required with this technique. Moreover, the use of organic solvents or harsher conditions induces fibrinogen denaturation or even amyloid formation.^16^ Recently, Hense et al. presented fibrin-like fibers that were formed from a fibrinogen solution without thrombin.^17^ They used low/moderate concentrations of multivalent oxoanions (corresponding to multiply deprotonated acids) such as phosphate and citrate at low temperature (5 °C) with sodium as a counterion.^17^ The resulting fibers resembled the native fibrin structure. Similarly to Hense et al., Galanakis et al. reported the formation of fibrin fibers from solutions enriched with soluble fibrin after precipitating fibrin at 4 °C and low sodium phosphate concentration.^18,19^ Nondenaturing buffers, thus, facilitate the most promising and cost-effective procedures to prepare fibrin-like fibers from fibrinogen without thrombin *in vitro.*

We also introduced a process for fiber formation under nondenaturing buffer conditions and achieved nanofiber assembly in highly saline aqueous environments by adding phosphate-buffered saline (PBS) and a drying step.^20,21^ Dense nanofibers with dimensions resembling native fibrin and the networks prepared by Hense et al.^17^ were formed at high salt concentrations without thrombin-induced cleavage. Mild secondary structure changes were found after salt-induced fibrinogen self-assembly without any amyloid transitions.^21^ Such 3D-fibrinogen networks presented good mechanical stability when hydrated, supported the cocultivation of fibroblasts and keratinocytes and prevented *E. coli* infiltration.^22,23^ They also enhanced platelet adhesion while minimizing their procoagulant activity and can therefore be used to steer blood-materials interactions.^24^

To understand which other salts may trigger fibrinogen fiber formation, we also studied the influence of divalent ions such as Ca^2+^ and Mg^2+^ on fibrinogen. While various monovalent ions drive^17,20^ or assist^25^ fiber formation, divalent ions did not induce fiber assembly in highly saline formulations, although they yielded much stronger changes in fibrinogen secondary structure than monovalent PBS.^26^ We showed that fibrinogen assembly into nanofibers could be induced by monovalent cations in direct contact with the protein, while multivalent cations did not interact specifically with solvent-exposed moieties of fibrinogen.^26^ Hence, we hypothesized that the primary hydration shell of monovalent cations is more loosely coordinated than that of divalent cations and that water ligands can be partly detached in contact with certain fibrinogen domains.^26^ So far, nanofibers were observed after drying fibrinogen with sodium or potassium phosphate and, to some extent, with sodium or potassium chloride.^20^ However, for fibers formed with those pristine salts, no further characterizations of their composition or internal structure have yet been presented.

To establish self-assembly of fibrinogen nanofibers as a reproducible process to prepare large-scale scaffolds for tissue engineering applications with controlled molecular structure, nanoarchitecture, and cell-binding properties, it is crucial to understand how specific monovalent ions influence this process. Integrating these influences into a constituting rule, such as a Hofmeister series for fibrinogen fiber formation, is essential. Furthermore, considering the biological relevance of monovalent ions, particularly those present in blood plasma, is vital. Therefore, this study aims to understand the influence of different monovalent cations, anions, and polyvalent anions on fibrinogen fiber assembly during drying in highly saline aqueous formulations. For the first time, we elucidate how the fibrinogen nanofiber assembly can be systematically tailored through specific kosmotropic anion–cation combinations. Drawing on this extended Hofmeister concept, our novel approach aims to enable the rational design of a salt-driven fibrinogen nanofiber assembly, which is of high relevance for biomaterial design.

## 2 Materials & Methods

### 2.1 Substrate Preparation

Round glass slides (VWR, Darmstadt, Germany) with a diameter of 12 mm were used as substrates for fibrinogen self-assembly^26^ and cleaned for 5 min by immersion in a mixture of 3:1 sulfuric acid (VWR) and 30% hydrogen peroxide solution (VWR). Subsequently, the slides were thoroughly rinsed and kept in deionized water from a TKA water purification system (Thermo Fisher Scientific, Schwerte, Germany). Before the self-assembly of fibrinogen fibers, the glasses were dried with nitrogen. For light scattering and FTIR analysis, cleaned glass slides were coated with a gold layer by using an EM ACE600 high vacuum sputter coater (Leica Microsystems, Wetzlar, Germany). First, an adhesion layer of 5 nm of chromium was applied, followed by 25 nm of gold. For scanning electron microscopy (SEM) investigations, cleaned glass slides were modified by immersion in an ethanolic (Honeywell, VWR) solution containing 5% (3-aminopropyl)­triethoxysilane (APTES, Sigma-Aldrich, Steinheim, Germany). Before subsequent modification with fibrinogen, APTES-modified glasses were washed with ethanol and dried with N_2_.

### 2.2 Buffers and Salt Solutions

To directly compare the results obtained for divalent metals with monovalent metals, in the current study, we used Tris as a background buffer, as in our previous report.^26^ All buffers and salt solutions were prepared using deionized water from a TKA water purification system. The pH was monitored and adjusted with a pH meter (Carl Roth GmbH, Karlsruhe, Germany) by adding concentrated solutions of HCl (VWR) or NaOH (VWR) dropwise, as required. Buffer solutions of Tris (Tris­(hydroxymethyl)-aminomethane (C_4_H_11_NO_3_, Roth) were prepared with a concentration of 10 mM and pH 7.0. Phosphate-buffered saline (PBS) (Gibco, Thermo Fisher) solution was used in a concentration of 5× and pH of 7.4. Sodium phosphate buffer with 300, 200, and 100 mM concentrations was prepared by mixing NaH_2_PO_4_ and Na_2_HPO_4_ (Carl Roth GmbH) solutions in different ratios (abbreviated as Na-PO_4_ from now on) to the desired pH of 7.0 in ultrapure water. The same concentrations and pH were prepared for potassium phosphate by mixing KH_2_PO_4_ and K_2_HPO_4_ (Sigma-Aldrich), abbreviated as K-PO_4_. Stock solutions of sodium chloride (NaCl, VWR) and potassium chloride (KCl, Carl Roth GmbH) were prepared with concentrations of 750, 1500, and 2250 mM in 10 mM Tris buffer, pH 7.0. Salt solutions of sodium acetate (C_2_H_3_O_2_Na_,_ Honeywell Fluka, Beilstein, Germany), sodium sulfate (Na_2_SO_4,_ Sigma-Aldrich), sodium citrate (C_6_H_5_O_7_Na_3,_ Sigma-Aldrich), magnesium sulfate (MgSO_4_•7H_2_O, Merck KGaA), and potassium sulfate (K_2_SO_4,_ Merck KGaA) were prepared with concentrations of 200 mM by dissolving the salts in ultrapure water. Likewise, a disodium oxalate (C_2_O_4_Na_2_, Merck KGaA) solution was prepared with a stock concentration of 100 mM.

### 2.3 Fibrinogen Stock Solution and Fibrinogen Self-Assembly

Fibrinogen stock solutions were prepared by dissolving 5 mg/mL of fibrinogen from human plasma (Merck KGaA, Darmstadt, Germany) in 10 mM Tris, followed by overnight dialysis using a 14 kDa cutoff cellulose membrane dialysis tubing (Sigma-Aldrich) to remove low molecular weight compounds.^20^ Fibrinogen precipitation was carried out by pipetting 60 μL of the fibrinogen stock solution and 60 μL of the respective salt solution on glass or gold substrates, respectively. For planar, i.e., nonfibrous, reference samples,^20^ instead of salt solutions, 60 μL of deionized water was added. Sample drying was performed overnight in a home-built humidity chamber at 24 °C and 30% relative humidity. Subsequently, the dried samples were placed in a Petri dish containing one microliter of 37% formaldehyde solution (FA, AppliChem GmbH) per cm^3^ and covered with parafilm. Following cross-linking in formaldehyde vapor for 2h, samples were aired in the fume hood for 30 min and rinsed with deionized water for 30–60 min (exchanging the water every 5 to 15 min), which removed any residual salt crystals.

### 2.4 *In Situ* Analysis of Fibrinogen Fiber Assembly

To monitor the drying process of selected fibrinogen samples in real-time, we utilized an optical scattering sensor device, the OS 500 from Optosurf (Ettlingen, Germany) in conjunction with a Sartorius TE 3102 (Göttingen, Germany) or a Kern TGD 50–3C pocket balance (Kern & Sohn, Balingen, Germany) mass balance, as previously described.^26^ To maintain a consistent humidity level during the *in situ* measurements, a continuous flow of dry air was introduced into the chamber. The light scattering sensor was positioned at a distance of 5 mm from the substrate surface, and a light-emitting diode with a wavelength of 676 ± 1 nm was used, generating a focused spot with a width of 0.9 mm that was centrally aligned on the substrate. By analyzing the light scattering patterns, we obtained data on both the overall intensity and the variance of the angle distribution (termed Aq roughness) of the detected light for each fibrinogen-coated surface. More details on the Aq parameter can be found in our previous report.^26^ First, baseline signals were established by characterizing gold samples without liquid deposition. Subsequently, aqueous fibrinogen and salt solutions were applied, and we continuously monitored time-dependent changes in intensity, mass, and Aq of the fibrinogen precipitates until the samples reached a completely dry state.

### 2.5 Morphological and Roughness Characterization

Roughness maps of cross-linked and washed fibrinogen samples were obtained with the same light scattering sensor device OS 500 used during *in situ* analysis at a measuring distance of 5 mm from the substrate surface, and again, a light-emitting diode (wavelength of 676 ± 1 nm) was used. Local roughness findings obtained by scanning a 30 μm light spot over the sample surface were integrated in an Aq map, i.e., a two-dimensional array of measuring spots, with a lateral distance of 50 μm between neighboring measurement regions, resulting in a total map width of 20 × 20 mm^2^. Roughness information on fibrinogen precipitates was averaged across the 30 μm light spots and several 100 μm wide regions. The overall surface appearance and substrate coverage were analyzed with macroscopic images taken with a Keyence VHX-7000 digital microscope (Keyence, Neu-Isenburg, Germany). The samples were imaged using 20× magnification in an overall survey, applying stitching for single partial images.

After cross-linking and washing of the precipitated fibrinogen, the morphology of the samples at the nanoscale was examined by SEM following sputter coating with a 7 nm-thick gold layer by using our EM ACE600 high vacuum sputter coater. For SEM imaging, we used a Phenom XL Desktop SEM (Phenom-World BV, Eindhoven, Netherlands) at acceleration voltages of 10 kV, employing the secondary electron detector. Fiber diameters were manually analyzed with ImageJ using a total of 50 measurements equally distributed on selected SEM images with 15,000× magnification. The position of each measurement region within a survey image is given in the Supporting Information (see Table S1).

### 2.6 Secondary Structure Analysis

To analyze the secondary structure of dried fibrinogen precipitates on gold, we utilized a Bruker Vertex 70 infrared spectrometer with IR Scope II, following our previous routine.^21,26^ Fourier-transform infrared (FTIR) spectra were recorded at 10 to 15 different positions, with a resolution of 4 cm^–^^1^ and 64 scans per measurement. At least three samples of each fibrinogen-salt combination were measured. All spectra were processed by using the OPUS software package provided by Bruker. The positions of the amide bands were determined by peak integration using the Origin 2020 software from OriginLab Northampton. The secondary structure, i.e., the amount of alpha helices, beta-strands, or other structures, was obtained by deconvoluting the amide I band using our previous procedure.^21,26^

### 2.7 Chemical Composition of Fibrinogen Precipitates

The surface composition of dried, cross-linked, and washed fibrinogen precipitates underwent characterization by X-ray photoelectron spectroscopy (XPS) using a Thermo Scientific K-Alpha X-ray Photoelectron Spectrometer with monochromatic Al K_α_ X-ray irradiation. The electron analyzer worked in Constant Analyzer Energy mode (CAE) with 150 eV for overview spectra, 40 eV for detailed scans in the Na 1s, K 2p, Cl 2p, S 2p, and P 2p spectral regions, and 20 eV for energetic high-resolution line spectra. The applied diameter of the area of analysis was 0.4 mm. The achieved sensitivity depended on the respective elements and allowed for detecting sodium at concentrations as low as 0.1 at. % within the information depth of several nanometers. All fibrinogen samples were analyzed after 30 min of cross-linking and rinsing with ultrapure water. Our previous rinsing procedure^26^ was modified by changing the water every 5 min to effectively remove salt deposits. To ensure comparability with our previous results,^26^ samples freshly prepared from 2.5× PBS were analyzed after they had been rinsed with the modified procedure.

All data obtained from the spectra were fitted by using the CasaXPS software (Version 2.3.19PR1.0, Casa Software Ltd.). To compensate for electrostatic charging effects, the C 1s photoemission line related to hydrocarbonaceous C–C/C–H-species was attributed a binding energy of 285 eV during data postprocessing, and all other lines were shifted using the same increment. Signal areas were obtained by performing Shirley background subtraction. During the fitting process, complex signals were deconvoluted using a sum of peaks with different positions and a Gaussian–Lorentzian line shape (GL(30)).

### 2.8 Molecular Dynamics (MD) Simulations

The input structure of the Human Fibrinogen D domain (Fg-D) was obtained from the Protein Data Bank (PDB) with the PDB code 1LT9.^27^ Fg-D is a monomer consisting of three chains: α, β, and γ. The α chain includes residues 126 to 190, the β chain includes residues 161 to 458, and the γ chain includes residues 96 to 394. The structure contains eight disulfide bonds, three of which are interchain bonds and the remaining five are intrachain bonds. The simulations were conducted at pH 7, with the protonation states of the titratable residues determined using the H++ 4.0 Web server.^28–30^ At this pH, Fg-D has a total charge of −1e, with e referring to the elementary charge. To study the binding of ions, we performed simulations of Fg-D including NaCl, KCl, MgCl_2_, Na_2_HPO_4_, and NaH_2_PO_4_ salts in the simulation cells at a concentration of 1.125 M, except for the Na^+^ concentration in Na2HPO4, which was set at 2 M.

The MD simulations were performed using the GROMACS software, version 2021.2.^31^ The AMBER14 force field was used to describe the bonding and nonbonding parameters, and the TIP3P model was used for the water solvent. The parameters for phosphate anions were taken from Kashefolgheta and Vila Verde.^32^ The CHARMM-GUI solution builder^33,34^ was used to prepare the GROMACS input files. The phosphate ions were inserted into the simulation box randomly using the “gmx insert” command. Analyses and graphical representation of the MD trajectories and snapshots were performed using the Visual Molecular Dynamics (VMD)^35^ and MDAnalysis.^36,37^ The input systems were first energy-minimized using the steepest descent algorithm until the forces converged to <1000 kJ/mol/nm to remove steric clashes. This was followed by NVT equilibrations at a constant temperature of 300 K, maintained using the V-rescale thermostat for 10 ns with position restraints on the backbone and side chain atoms of the protein. Subsequently, NPT equilibrations were conducted at a constant temperature of 300 K and pressure of 1 bar for 10 ns, maintained using the Berendsen barostat. The final production simulations were performed using the NVT ensemble for 100 ns.

### 2.9 Analysis of Protein–Ion Interactions

Three distinct analyses were performed to gain insight into the interactions between the Fg-D protein domain and the different ions.

#### 1. Distance Distribution Analysis

The first step involved quantifying the protein–ion contacts by calculating the distance distributions between specific ions (Na^+^, K^+^, Mg^2+^, Cl^–^, and HPO_4_^2–^) and the Fg-D domain. The distances were measured using the MDAnalysis.analysis.distances tool, excluding hydrogen atoms to focus on the core interactions. For the HPO_4_^2–^ ions, the distances were calculated from the phosphorus atom. From the distributions, we determined cutoff distances for ion-protein contacts, which were used in subsequent analyses.

#### 2. Ion Retention within Cutoffs

Using the determined cutoff distances, we analyzed the percentage of time that each ion remained in contact with the protein throughout the simulations. Ions that were found within the cutoff distance for >50% of the simulation time were classified as immobile ions and selected for further analysis. Additionally, a similar analysis was conducted on the Fg-D domain (excluding hydrogen atoms) to assess the percentage of time that each amino acid residue was in contact with any of the ions.

#### 3. Hydration Shell Analysis

The final step involved examining the hydration shells around the immobile ions. We employed the Radial Distribution Function (RDF) analysis tool in VMD to determine the probability *g*(*r*) of the ions being surrounded by water molecules at various distances *r*, averaged over the simulation time. The RDF was calculated between the immobile ions and the oxygen atoms of the water molecules. Integration of the RDF over the distance yielded the coordination number (CN), which offered detailed information about the number of water molecules surrounding each ion at specific distances.

## 3 Results and Discussion

In our previous studies, drying saline fibrinogen formulations in the presence of PBS or sodium phosphate solutions reproducibly resulted in well-defined fibrous structures on substrates from different materials.^20,21,26^ Minimum starting concentrations of 0.5× PBS or 5 mM Na-PO_4_ were necessary to induce self-assembly of fibrinogen nanofibers,^20^ with lower salt concentrations leading to a lower surface coverage with nanofibers. So far, the best fiber coverage was achieved starting with 50 mM Na-PO_4_ and 2.5× PBS, equivalent to 375 mM NaCl.^20^ Therefore, we have now used these concentrations as a starting point to investigate fibrinogen precipitation in the presence of NaCl, KCl, Na-PO_4_, and K-PO_4_ and also studied the influence of higher starting concentrations of monovalent salts.

### 3.1 Precipitation of Fibrinogen Is Altered in the Presence of Different Monovalent Salts

As established for divalent salts and PBS,^26^ we used angle-resolved light scattering to monitor the Aq roughness of the solid below the droplet as a dynamic indicator of surface topography during fibrinogen precipitation until complete evaporation of water (see Figure 1). The time-dependent Aq roughness curves were obtained at the center position in a 0.9 mm wide spot while fibrinogen was dried with different monovalent salts at varying concentrations. We used 2.5 mg/mL fibrinogen with varying concentrations of sodium or potassium chloride (375, 750, and 1125 mM) and sodium or potassium phosphates (50, 100, and 150 mM) before drying. In Figure 1, low Aq values indicate a very smooth surface and small roughness, while higher Aq values correspond to higher roughness. At 0 h, the initial roughness corresponds to the underlying gold substrate before adding fibrinogen and salt solution. After depositing a transparent droplet composed of salt, Tris, and fibrinogen, a 30 min initial equilibration phase of relative humidity was observed, followed by an ongoing mass loss, which was attributed to water evaporation during drying (see Figure S1 in the Supporting Information). Based on the mass variation and calculated solution concentration, the arrows in Figure 1 indicate when the fibrinogen-containing droplets reached the salt solubility limit, at which point salt crystals are expected to precipitate. Moreover, we marked when the maximum solubility of the fibrinogen used in this study, i.e., 10 mg/mL in Tris buffer, would be reached, based on mass and water evaporation. Salt solubility was typically reached after 80% of the water evaporated. Overall, we observed variations in the drying time that ranged from 3 to 5.5 h, depending on the salt type and concentration. Fibrinogen solutions with phosphates dried faster than those with chloride, although solid chlorides are less hygroscopic. Generally, higher salt concentrations increased the drying times due to a reduction in water vapor pressure. In the first 2 h, significant mass loss occurred with minimal change in Aq values (around 5 or below), indicating no precipitation or a very smooth layer. This suggests that fibrinogen remains dissolved during the initial phase and does not yet contribute to surface structuring detectable by Aq. After 2 h, Aq values rose, and solutions became turbid, which coincided with the start of the rise in Aq values. The simultaneous increase in turbidity and roughness strongly suggests that fibrinogen begins to aggregate and precipitate onto the substrate surface, contributing directly to the rise in Aq. The shape of the Aq curves varied depending on the salt concentration and type, with chloride and phosphate salts showing similar features within their groups.

**Figure 1:**
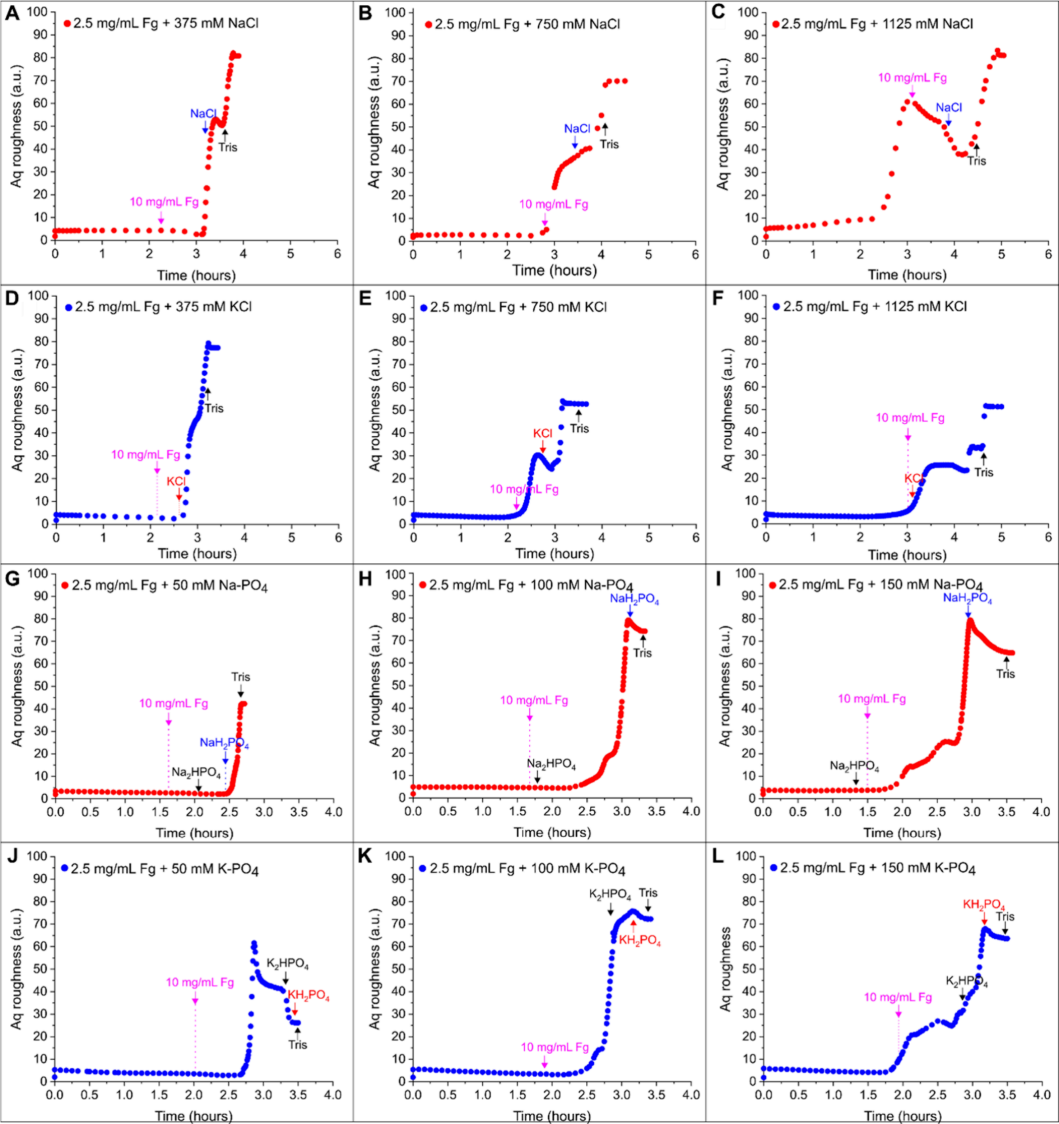
In situ monitoring of the surface roughness of dried fibrinogen precipitates. Fibrinogen droplets containing 2.5 mg/mL fibrinogen in 5 mM Tris were dried at increasing concentrations of (A–C) NaCl, (D–F) KCl, (G–I) Na-PO_4_, and (J–L) K-PO_4_. Aq versus time curves were obtained from light scattering analysis of fibrinogen (Fg) precipitation on gold in the presence of different monovalent salts. In each plot, the round dots represent data points (in red or blue color for salts containing Na^+^ or K^+^, respectively) measured until complete sample drying and indicate the change in Aq surface roughness during the drying of the different fibrinogen-salt solutions. The initial Aq values at 0 h represent the roughness of the underlying gold substrate before the fibrinogen and salt solutions were added. The different arrows mark the time point when the respective salts are expected to reach their solubility limits (following a concomitant gravimetrical assessment) and precipitate.

As shown in Figure 1, the overall change in Aq during drying (Δ­(Aq)_total drying_) was mostly above 40, reaching up to 90 for fibrinogen with various monovalent salts, except for lower concentrations of K-PO_4_ (50 mM), which reached only an Aq of 26. Aq vs time curves for fibrinogen-free salt solutions only showed minor increases in Aq during drying, with overall Δ­(Aq)_total drying_ values between 2 and 17, and for Na-PO_4_ and K-PO_4_ even below 10 (see Supporting Information, Figure S2), indicating smoother surfaces without fibrinogen. This is in contrast to divalent chlorides that previously exhibited a Δ­(Aq)_total_drying_ between 20 and 70 when dried without fibrinogen.^26^ The low Aq values for monovalent salts in the sample center also correlate well with optical microscopy images of NaCl and KCl precipitates, which showed that the sample center was mostly free of large salt crystals (see Figure S3 in the Supporting Information).

To understand whether fiber formation occurred close to the salt solubility limit or rather near the complete sample drying point, we increased the initial salt concentration and analyzed the slope of the first Aq increase in the Aq vs time plots. This salt increase allowed us to separate the respective onset times of fibrinogen and salt precipitation during our *in situ* analysis. In our reference measurements of both chlorides, starting with 375 mM, and both phosphates, starting with 50 mM, we did not observe any notable Aq increase during drying (see Supporting Information, Figure S2). For fibrinogen with 375 mM NaCl or KCl, the Aq roughness showed two steep slopes: the first coinciding with salt saturation and the second with Tris saturation (see Figure 1A,D). Initially, Aq was below 10 and constant until 2.5–3.0 h, before it sharply increased to around 80 for both salts. With 750 mM chlorides, a less steep two-step Aq rise occurred, reaching final values of 70 for NaCl and 55 for KCl (see Figure 1B and E). For both lower concentrations, the saturation of the salts was reached during the initial changes of the Aq values. At 1125 mM, an additional step appeared between the two already present at lower chloride concentrations, with final Aq values around 85 for NaCl and 55 for KCl (see Figure 1C,F). KCl showed a continuous three-step increase, while NaCl had a decrease between the salt and Tris saturation events.

For fibrinogen and alkali phosphate salts, Aq changes were similar to those for chlorides but with curves limited to a narrower time window. Na-PO_4_ and K-PO_4_ solutions contain mixtures of phosphates with different solubilities (NaH_2_PO_4_ and Na_2_HPO_4_ or KH_2_PO_4_ and K_2_HPO_4_) with differently protonated anions and their hydrates showing different solubilities,^38,39^ causing salts to precipitate at different drying times (see arrows in Figure 1G–L). At 50 mM, Na-PO_4_ and K-PO_4_ with fibrinogen showed distinct Aq vs time curves. Initially, Aq was low and constant until a sharp increase at 2.0–2.5 h occurred in less than 30 min. For Na-PO_4_, Aq increased when NaH_2_PO_4_ started precipitating, while Na_2_HPO_4_ precipitation caused no change. However, we did not observe any corresponding change in the Aq value. This may be attributed to the presence of a pH-dependent mixture of different salts at subsaturation concentrations. For K-PO_4_, the Aq increased earlier than expected and did not coincide with the expected precipitation of any of the phosphate salts. It decreased in two steps, likely due to less soluble hydrates of K_2_HPO_4_ and KH_2_PO_4_.

Starting with 100 and 150 mM, both fibrinogen-phosphate mixtures had similar Aq curves. A small shoulder appeared at the initial Aq rise for 100 mM, followed by a steep rise, reaching Aq values of 80 during 1 h, then decreasing to 75 at the final drying point for both phosphates. Starting with 150 mM, another shoulder appeared, and the curve was less steep, taking 1.5 h to reach a final Aq of 65. For Na-PO_4_, Na_2_HPO_4_ precipitation was expected before the Aq rise, with NaH_2_PO_4_ precipitation coinciding with the Aq increase. However, starting with higher concentrations of K-PO_4_ showed Aq changes correlating well with the expected precipitation of K_2_HPO_4_ and KH_2_PO_4_.

Overall, *in situ* monitoring of drying fibrinogen solutions revealed that mutual interactions are present during salt and fibrinogen coprecipitation that influence the roughness of the dried precipitates. Comparing all graphs in Figure 1, Aq further increased at the end for NaCl and KCl, when Tris was expected to precipitate, but decreased for the phosphates. This is because Tris forms tris-hydrochlorides that have a rough texture, as we reported previously,^40^ causing the Aq profiles to increase. With phosphates, Tris-HCl did not precipitate, resulting in a decreased roughness. Since fibrinogen saturation was expected before salt saturation, apart from the mixture of 2.5 mg/mL fibrinogen with 150 mM Na-PO_4_, our findings suggest that starting from lower salt concentrations, major changes in Aq roughness are linked to the onset of salt precipitation. Increasing turbidity during drying indicates protein aggregation and precipitation,^20,41^ possibly due to a lower solubility threshold of fibrinogen that might correspond to a salting-out effect in a high-saline environment. This could mean more salt, i.e., a higher ionic strength, decreases fibrinogen solubility,^42^ bringing the Aq increase event closer to the 10 mg/mL saturation limit, suggesting early coprecipitation with salts. The resulting surface roughness thus serves as an indicator of the time and extent of fibrinogen precipitation during drying. However, increasing the initial salt concentration showed changes in Aq profiles not directly related to salt precipitation, indicating fibrinogen precipitation before the salts. In a nutshell, our time-dependent combined assessment of the Aq roughness of the deposits and the mass loss of the droplet during drying suggests that not only the final phase but rather an essential period of the precipitation process after the onset of a roughness increase is dominated by coprecipitation of fibrinogen and the respective salts. Validation of this hypothesis requires further analysis of salt-dependent fibrinogen precipitation, including turbidity measurements, as saturation levels may vary with solution composition and the resulting ionic strength.

### 3.2 Variations in Fibrinogen Morphology and Roughness Depend on the Monovalent Salts Present during Drying

To study whether high surface roughness correlates with fiber formation, as we previously observed for fibrinogen drying with PBS in comparison to divalent salts or Tris,^26^ we combined Aq mapping with optical microscopy and SEM analysis of cross-linked and washed fibrinogen precipitates obtained from solutions with different monovalent salts (see Figure 2). The macroscopic features found in optical microscopy images of all fibrinogen precipitates correlated precisely with the roughness information in the Aq maps, where high Aq values (red) indicate high surface roughness, while low Aq values (blue) indicate smooth regions.

**Figure 2:**
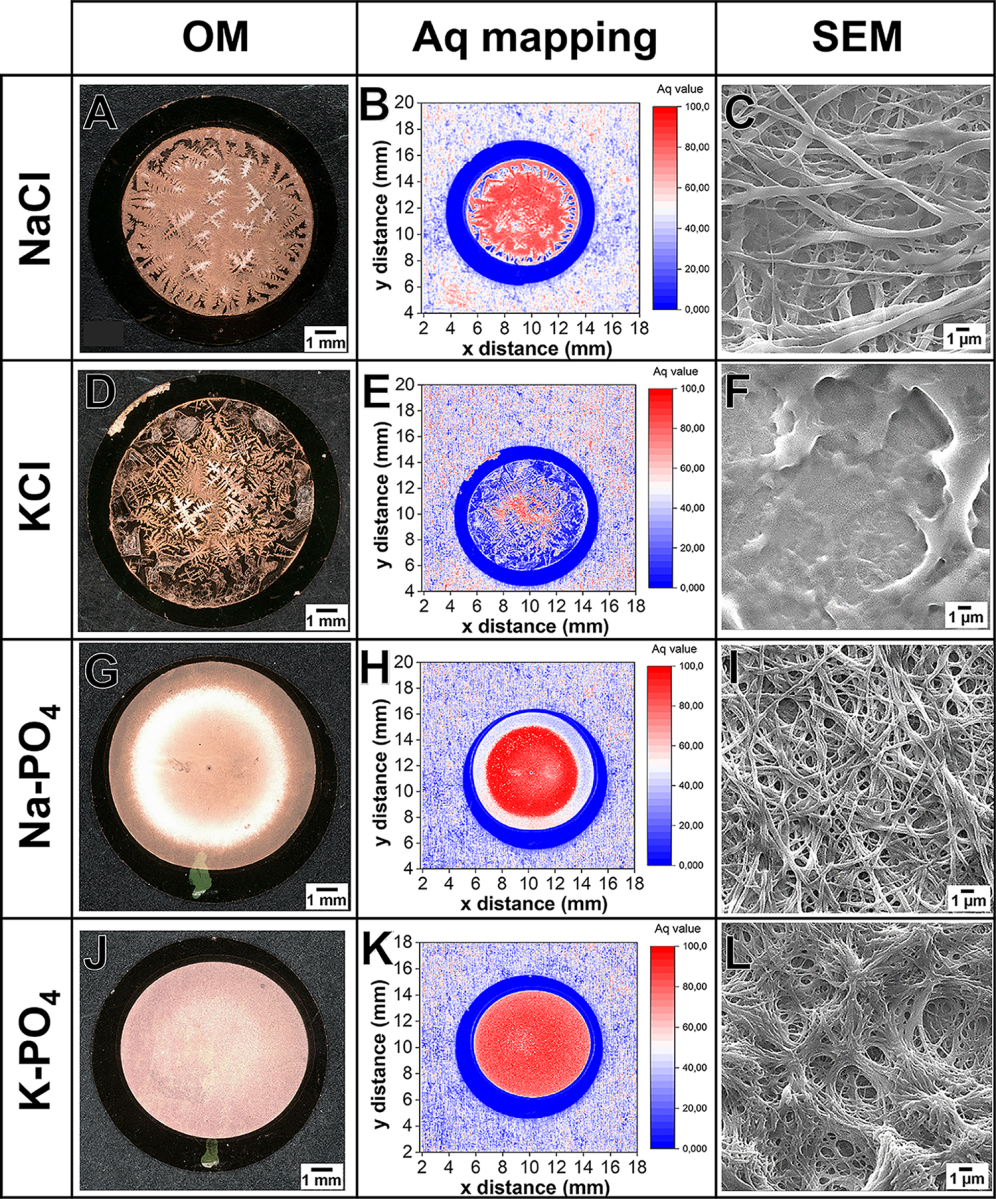
Morphology and roughness of fibrinogen dried in the presence of different monovalent salts. Optical light microscopy (OM), Aq-based survey images and SEM-based local detail images of samples prepared after drying 2.5 mg/mL fibrinogen in the presence of different salts: (A–C) 375 mM NaCl, (D–F) 375 mM KCl, (G–I) 100 mM Na-PO_4_, and (J–L) 100 mM K-PO_4_. For SEM analysis, all precipitates were prepared on APTES-modified glass, while for Aq mapping, fibrinogen films were prepared on gold. Light microscopy views correspond to Aq mapping with rougher areas (red color) being an indication of potential fiber formation. SEM images reveal (C) low density, highly coalesced fiber formation for NaCl, (F) no fiber assembly for KCl, (I) high density fiber assembly for Na-PO_4_ and (L) assembly of partly coalesced fibers with high density for K-PO_4_.

Fibrinogen precipitated starting with 375 mM NaCl showed dendritic features of 0.1 to 0.2 mm width with high roughness and an average Aq value around 75 (see Figure 2A,B and Figure S4). Similar dendrites were previously observed when fibrinogen, collagen, or BSA were precipitated in the presence of NaCl.^40,43^ SEM analysis of fibrinogen starting with 375 mM NaCl now revealed nanofibrous deposits only in a few distinguished sample regions (see Figure 2C and Figure S5A–D). Remarkably, those regions were surrounded by elevated areas that appeared rough but did not show a nanofibrous morphology. Detailed analysis revealed domains with flat and apparently smooth fibrinogen layers (seeFigure S5A, blue arrow) and domains with rough but not fibrous structures (see Figure S5C,D). Comparing our previous SEM analysis of unrinsed fibrinogen-NaCl precipitates (see Figure S6A^40^) with the SEM images of washed fibrinogen precipitates after Tris and NaCl crystals had been dissolved (see Figures S5A and S6B), we conclude that fibers had formed underneath the largest branches of the salt dendrites, which consist of crystallites with a width of around 200 μm. Planar fibrinogen regions were associated with areas between the NaCl dendrites. Interestingly, below small NaCl crystals, fibrinogen showed a rough but not fibrous morphology, contrasting with previous findings where PBS or Na-PO_4_ precipitates yielded fibers around and on top of salt crystals.^20^ Fibrinogen layers formed starting with 375 mM KCl were shaped by simultaneous salt dendrite formation (see Figure 2D) with an average Aq roughness of 40 that was much lower than with NaCl (c.f. Figure 2E,B). The highest Aq roughness was detected in the central sample region with the lowest KCl dendrite density. These fibrinogen-KCl precipitates had a thickness of at least 0.1 mm following cross-sectional analysis with 3D-optical microscopy (see Supporting Information, Figure S7). Interestingly, no fibers were found with KCl—neither around nor under KCl crystals (see Figure 2F). Detailed SEM analysis revealed both smooth and clumpy domains (see Supporting Information, Figure S5G,H), which matches Aq mapping results, showing a less rough surface than fibrinogen films formed from saturated NaCl solutions. Notably, in our previous study, we reported fiber formation starting with 375 mM KCl, but fibers were less defined than those from PBS salt solutions and distributed differently.^20^ The previous study used NH_4_HCO_3_ instead of Tris for dialyzing fibrinogen. NH_4_HCO_3_ decomposes into volatile gases and water during drying,^44^ while Tris is nonvolatile with a lower pH^45^ and therefore still present at the end of drying. These differences could indicate an influence of the pH value and the ions in the background buffer on the course and completion of fiber formation upon drying of the fibrinogen solutions. Further studies are required to confirm this hypothesis.

Figure 2G–I shows that fibrinogen precipitated starting with 100 mM Na-PO_4_ had rough, homogeneous surfaces with Aq values up to 100, correlating well with microscopic details. Light microscopy showed a flat surface without large salt crystals, but Aq mapping revealed high roughness. SEM analysis after rinsing linked this roughness to dense fibrinogen nanofibers, evenly spread over the surface (see Figure S5E,F). Similar results were seen starting with 100 mM K-PO_4_, but these fibers appeared more coalesced (see Figure 2J–L and Figure S5I,J). Overall, Na-PO_4_ resulted in the highest fiber formation and coverage with no large salt crystals or gaps observed. Our combined analysis suggests that higher roughness (Aq_max_ ≥ 80) indicates fiber assembly, especially compared to previous findings with divalent salts, where low Aq values (Aq_max_ ≤ 80) corresponded to no fiber formation.^26^

To analyze the contribution of salt crystals to film roughness, we additionally compared Aq values of droplets directly after drying with those after cross-linking and dissolving salts and Tris by washing. Before cross-linking and rinsing, Aq values were 80.8 for NaCl, 77.3 for KCl, 74.2 for Na-PO_4_, and 72.3 for K-PO_4_, matching the average Aq roughness in 1 mm regions around the center of cross-linked and washed fibrinogen precipitates (see Supporting Information, Figure S4). Aq values slightly decreased for NaCl and KCl after washing, while they slightly increased for Na-PO_4_ and K-PO_4_. This suggests chloride crystals add roughness on top of fibrinogen (see Figures S3 and S4), while phosphate crystals, that are smaller than chloride crystals, fill valleys in the undulated fibrinogen topography.^22,24^ Differences in Aq values before and after rinsing were within the standard deviation, indicating that the roughness was mainly due to fibrinogen content.

For future applications in tissue engineering, lateral scaffold homogeneity is crucial. Therefore, we analyzed the average <Aq> value and standard deviation of the Aq roughness in a 1 mm wide central region of cross-linked and washed fibrinogen films presented in the center column of Figure (and see Figure S4). The lowest values were found for KCl (68.2) and NaCl (77.9). Na-PO_4_ and K-PO_4_ had higher, similar <Aq> values (81.7 and 81.3). The highest standard deviations in the central region were observed for NaCl and KCl, indicating the highest variation in Aq mapping. This lower homogeneity for chloride salts is attributed to their larger crystal size and potential gaps in water-rinsed films (c.f. Figure 2, Figures S5 and S6). Moreover, KCl yielded no fibers, and NaCl fibers were not uniformly distributed, with gaps filled by salt crystals before rinsing (see Figure S6). Hence, monovalent salts with phosphates will be best suited to produce nanofibrous fibrinogen scaffolds for regenerative medicine in the future.

An important parameter to steer cell-scaffold interactions is the diameter of individual fibers in the scaffold. Therefore, we analyzed average fiber diameters from SEM images (see Figure S8 and Table S1) and found the lowest diameter of 228 ± 49 nm after drying fibrinogen starting with 100 mM Na-PO_4_, followed by 233 ± 67 nm for 100 mM K-PO_4_ and 301 ± 76 nm for 2.5× PSB. Starting with 375 mM NaCl, only a few regions with fibers were found that yielded an average diameter of 371 ± 131 nm. These results show that more fibrinogen was converted into persistently thin fibers with phosphates than with chlorides (cf. Figure 2). In particular for K-PO_4_, the coalesced fibers were also prone to some Ostwald ripening effect.^46^ We suggest that material exchange between neighboring fibers may be ruled by a salt-dependent transport-related barrier that counteracts coalescence with the following order: KCl < NaCl < K-PO_4_ < Na-PO_4_.

Recently, Hense et al. studied the effect of different salts on fibrinogen fiber formation at lower concentrations (15 mM) than we used.^17^ Interestingly, in their study, NaCl was considered to hinder fiber formation, while Na-PO_4_ promoted fiber formation, similar to our findings with protonated phosphates using sodium or potassium alkali counterions. They focused on sodium salts at pH 7.0, concluding that oxygen-containing, multivalent anions like phosphate or citrate best support fiber formation. Wei et al. previously also observed how ions in PBS influence fibrinogen aggregation and fiber assembly under acidic conditions at pH 2, noting increased fiber yield in the presence of PBS.^25^ Based on our findings, we conclude that fibrinogen precipitates into different fibrous or nonfibrous morphologies under varying environmental conditions, indicating that specific ion-protein interactions mediate fibrinogen aggregation and consequently fiber assembly.

Moreover, we studied the effect of higher starting concentrations of salt on fibrinogen precipitation. We obtained Aq maps and SEM images similar to those starting with lower salt concentrations for all salts (c.f. Figures S9 and S10 with Figure 2). Higher NaCl and KCl concentrations did not lead to an increase in surface roughness or coverage, nor did they promote fiber formation in the presence of KCl. For phosphates, fibrinogen coverage increased from 50 to 100 mM but remained unchanged at 150 mM, with no change in the fiber morphology. Therefore, we focused our further analyses of ion uptake and secondary structure on the lowest starting salt concentrations providing sufficient coverage: 375 mM for KCl and NaCl, and 100 mM for K-PO_4_ and Na-PO_4_.

### 3.3 Ion Uptake Is Stronger for Na^+^ and Only Observed for Fibrous Fibrinogen

Previously, for fibrinogen dried with PBS we observed that sodium cations were present in washed fibrinogen fibers while divalent cations were completely rinsed away.^26^ Hence, we hypothesized that ion-protein interactions may be affected by the hydration shells of both the ions and the protein and that sodium ions were bound to anionic sites within fibrinogen.^26^ To confirm this hypothesis, we now analyzed the elemental surface composition of cross-linked and rinsed fibrinogen precipitates that were dried with different monovalent salts (375 mM NaCl or KCl and 100 mM Na-PO_4_ or K-PO_4_) using XPS.

The atomic surface concentrations from the XPS inspection are presented in Table 1. The nitrogen surface concentration [N] remained around 17 at. % for all samples, similar to our previous study.^26^ In the presence of NaCl and KCl, no metallic cation or Cl-based moieties were detected, suggesting that both chloride salts were washed away. Fibrinogen samples prepared with PBS showed some scattering and lower [Na]/[N] concentration ratios between 1.3% and 2.6% compared to the previously detected 4.9%.^26^ It is noteworthy that although the modified rinsing process dissolved not only salt deposits but also the sodium cations from the fibrinogen, the sodium itself was not completely rinsed out. We attribute the decrease in [Na]/[N] concentration ratio to the multiple washing steps in which we now exchanged water every 5 min instead of every 15 min.^26^ When we rinsed one of the samples that had a [Na]/[N] ratio of 2.6% after 30 min of rinsing for an additional 15 min (see Table 1, second row), we found that the concentration decreased by slightly more than half. This confirmed our hypothesis that Na^+^ can be leached away from the formed fibrinogen fibers despite being intimately associated with the fibers. We suppose that Na^+^ ions can be replaced by H^+^ ions from water following an ion exchange process. Although PBS also contains KCl and K-PO_4_ in its formulation, we did not detect potassium species in either of the rinsed samples, agreeing well with our previous results.^26^ Moreover, XPS results showed no Tris residues on the surface, in neither overall nitrogen surface concentration [N] nor in the contribution of C*O species to the C 1s signal. This agrees well with the Aq maps and SEM images (see Figure 2), which showed no clear indication of Tris salts after rinsing cross-linked and dried fibrinogen films.

**Table 1:** Composition of Fibrinogen-Salt Precipitates on APTES-Coated Glass Slides after Cross–Linking Fibrinogen with FA Vapor and Rinsing with Pure Water for 30 min Obtained from XPS Analysis and Given in Atomic % (at. %) in the Case of [N] and in % in the Case of Ratios Normalized to [N]^a^

sample	metal ion in starting solution	rinsing time (min)	[N]	[metal]/[N]	[Cl]/[N]	[S]/[N]	[P]/[N]
	Na^+^, K^+^			metal = Na			
Fg & 2.5× PBS pH 7	morphology: highly fibrous	30 min	14.7/17.5/17.2	4.9*/1.3/2.4	0.9/0/0	2.5/2.8/3	-/0.1/0.08
Time series rinsing for PBS (Na^+^ leaching)	morphology: highly fibrous	30 min 30 + 15 min	16.5/17.06	2.61.1	0/0	3.1/3.2	0.07/0.04
	Na^+^			metal = Na			
Fg & 375 mM NaCl, pH 7	morphology: low fibrous	30 min	17.5/17.7/17.3/17.3	0/0/0/ 0	0/0/0/ 0	2.9/2.6/2.8/2.4	0.1/0.2/0.08/0.1
	Na^+^			metal = Na			
Fg & 100 mM Na-PO_4_, pH 7	morphology: highly fibrous	30 min	17.2/17.0/17.6	1.2/1.0/1.1	0/0/0	3.1/2.7/2.7	0.09/0.2/0.06
	K^+^			metal = K			
Fg & 375 mM KCl, pH 7	morphology: rough with smooth domains; no fibers	30 min	17.7/16.5	0/0	0/0	2.8/2.5	0.1/0.1
	K^+^			metal = K			
Fg & 100 mM K-PO_4_, pH 7	morphology: highly fibrous	30 min	17.2/17.3	0/0	0/0	2.7/3.3	0.2/0.2

a __/__ represents one position of one measurement per sample. *, result from previous publication.^26^

Since fibrinogen precipitates prepared with NaCl did not show any Na^+^ after washing, we presume that for PBS samples, exchangeable sodium-fibrinogen moieties originated from dissolved sodium phosphate salt. Remarkably, XPS analysis of fibrinogen layers prepared with Na-PO_4_ showed a [Na]/[N] concentration ratio of about 1.2%, with no detectable phosphate species. This confirms our hypothesis that Na^+^ in fibrinogen-PBS samples originated from Na^+^ counterbalanced by (protonated) phosphate ions. Since K^+^ was not detected in fibrinogen-PBS samples, we did not expect any potassium in fibrinogen-K-PO_4_ either, which was confirmed by our XPS findings.

On the basis of these results, we hypothesize that nanofibrous fibrinogen preferentially captures Na^+^ rather than K^+^, preferentially in the presence of O-containing counterions in solution, such as (protonated) phosphates. Presumably, this preference is due to the smaller van der Waals radius of Na^+^ (227 pm) compared to K^+^ (275 pm).^47^ The preferential sorption of sodium over potassium on protein surfaces was studied by Vrbka et al.^48^ via molecular dynamics simulations for actin, bovine pancreatic trypsin inhibitor, ubiquitin, and ribonuclease. They suggested that interactions between the carbonyl oxygen of the amide groups and Na^+^ are responsible for this preference. We previously hypothesized that sodium ions can disrupt their hydration layer to establish direct protein contacts and be integrated into the protein structure.^26^ As the concentration of peptide groups exceeds the maximum detected Na^+^ concentration by more than 1 order of magnitude, we suggested carboxylate groups in fibrinogen side-chains as potential binding sites for monovalent cations.^26^ This hypothesis will be tested later in this study by means of all-atom molecular dynamics simulations (see Section 3.6).

Our SEM findings reveal fiber formation with K-PO_4_, even though K^+^ was not detected on thoroughly washed fibrinogen films. Possible explanations for the higher affinity of Na^+^ over K^+^ might lie in thermodynamic effects such as stronger cation-carboxylate interactions, possibly also via cooperative complexation with more than a single ion-anion pair,^49^ or kinetic effects such as reduced activation barriers in the cation/H^+^ exchange during rinsing. Since a stronger ion/protein complexation might be associated with more pronounced changes in secondary protein structure, we also analyzed fibrinogen films by means of vibrational spectroscopy.

### 3.4 Secondary Structure of Fibrinogen Precipitates Does Not Correlate with Fiber Formation

To study whether specific ion-protein interactions are associated with conformational changes, we conducted FTIR analysis of cross-linked and washed fibrinogen precipitates that were dried with NaCl, KCl, Na-PO_4_, and K-PO_4_ (see Figure 3).

**Figure 3:**
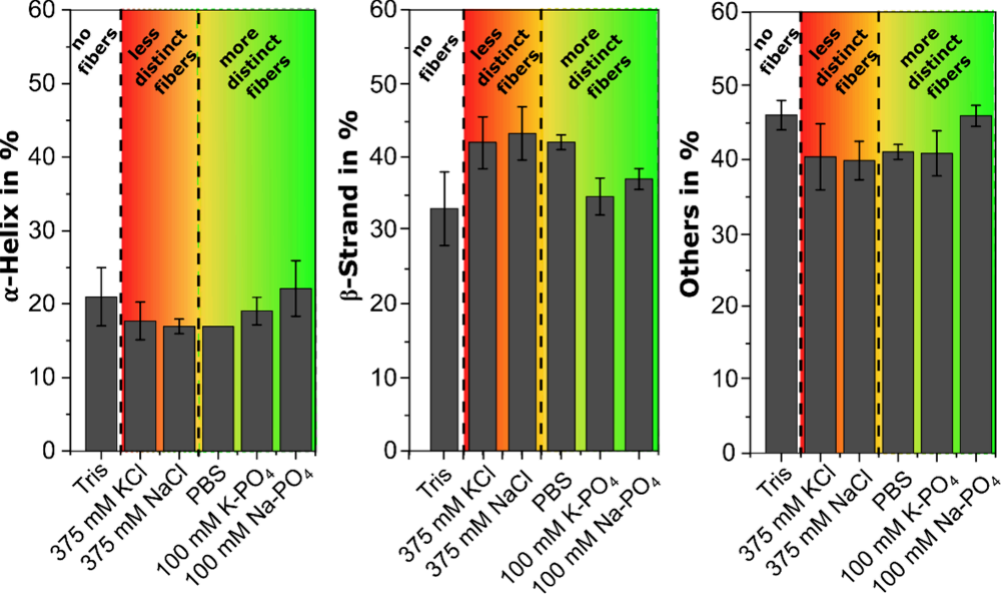
Secondary structure of fibrinogen precipitates. 2.5 mg/mL fibrinogen were dried with different monovalent salts, cross-linked and washed. The histogram presents the results from the deconvolution applied to the amide I bands of the FTIR spectra (average of *n* = 3 independently prepared samples). For comparison, the results for fibers assembled with PBS from our previous study^26^ are also shown. No clear correlation between secondary structure changes and fiber formation was observed.

The resulting FTIR histograms were ordered following the SEM-based structural characteristics, starting from nonfibrous fibrinogen films prepared in Tris (left) to the most nanofibrous films with Na-PO_4_ (right). The α-helix content decreased from smooth fibrinogen with Tris (21% ± 4%) via fibrinogen with KCl (18% ± 3%) and NaCl (17% ± 1%), which did not necessarily result in fiber assembly to fibrinogen fibers with PBS (17% ± 0%). The α-helix content was the highest for K-PO_4_ (19% ± 1%) and Na-PO_4_ (22% ± 4%), the most fibrous fibrinogen precipitates. β-strand content was lowest for Tris (33% ± 5%) and both phosphate samples (Na-PO_4_: 37% ± 1% and K-PO_4_: 35% ± 3%) and highest for PBS (42% ± 1%) and both chlorides (NaCl: 43% ± 4%, KCl: 42% ± 4%), all showing more β-strand than the smooth samples prepared with Tris. Despite the strong differences in morphology, fibrinogen with Tris and K-PO_4_ had the highest amounts of other structures, around 46%, while for all other salts, the percentage of other structures was between 40 and 41%. Overall, more fibrous fibrinogen precipitates assembled with phosphates had less β-strand and more α-helix content than the less fibrous chloride precipitates (c.f. Figures 3 and 4). PBS, which contains 10 times as much NaCl as phosphates, had a secondary structure that resembled NaCl rather than phosphates. This conformational similarity is also reflected by the similar fiber diameters obtained from SEM analysis (see Supporting Information, Figure S8).

**Figure 4:**
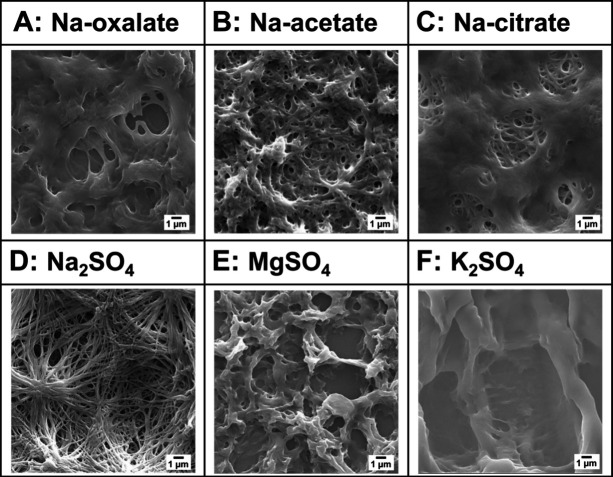
Morphology of fibrinogen precipitates dried with different polyvalent anions. SEM images of samples prepared after drying 2.5 mg/mL fibrinogen with different salts using the following starting concentrations: (A) 50 mM Na-oxalate, rough without fibers; (B) 100 mM Na-acetate, rough with fibrous features; (C) 100 mM Na-citrate, fiber-like structures between smooth domains; (D) 100 mM Na_2_SO_4_, fiber-like structure with star-shaped profile; (E) 100 mM MgSO_4_, rough without fibers; (F) K_2_SO_4_, smooth. Salts with Na^+^ are more favorable to form fibers than the ones with K^+^.

Overall, the secondary structure profiles show that both chlorides behave similarly to the addition of PBS to fibrinogen, for which we had previously reported a decrease in α-helix and an increase in β-strand content.^20,21^ We had suggested that the increase in β-strand structures in fibrous fibrinogen layers prepared with PBS correlated with a transition from smooth to fibrous layers.^21^ However, further studies with divalent ions have shown that an increase in β-strand content does not necessarily result in fibrinogen fiber assembly.^26^ This finding is corroborated here, since although there was an increase in β-strands for NaCl and KCl, the assembly of fibrinogen into fibers was limited or even suppressed. For fibrinogen with both phosphates, we also obtained similar conformational trends. Interestingly, these secondary structure profiles were close to that of native fibrinogen in aqueous solution as we had previously measured by CD spectroscopy (26% α-helices, 27% β-strands, and 47% other structures).^21^

Comparing results from divalent salts with monovalent salts, the β-strand content for phosphates matched that of divalent chlorides.^26^ This reinforces that secondary structure changes alone cannot explain why fibrinogen assembles into fibers with phosphate salts during drying or why fibers form inconsistently with monovalent chlorides. Additionally, it does not explain why monovalent chlorides yield some roughness, while divalent chlorides result in smooth layers. However, as discussed previously,^26^ FTIR findings can indicate or exclude fibrinogen denaturation during fiber assembly, which is important for the future use of these scaffolds in regenerative medicine. With our previous results^21,26^ and current findings, we confirm that the secondary structure changes of fibrinogen after assembly into fibers in the presence of certain monovalent salts are minor, similar to those observed after formation of fibrin from thrombin-activated fibrinogen.^50,51^

### 3.5 Fiber Formation with Polyvalent Anions Depends on Cation/Anion Pairing

So far, we have not detailed why fibrinogen is converted to better-developed fibers in the presence of phosphates instead of chlorides. The most defined fibers were observed with hydrogen phosphates ([HPO_4_]^2–^ and [H_2_PO_4_]^−^), which are polyvalent and amphoteric anions that act as both acids and bases and can form several hydrogen bonds and salt bridges.^52^ With reference to the Hofmeister series, several polyvalent anions, classified as kosmotropic anions, may have similar effects. To further understand the effect of pairing the monovalent cations Na^+^ and K^+^ with kosmotropic anions, we therefore studied the capability of different polyvalent anions to promote fibrinogen fiber formation.

First, we tested sodium sulfate and three different sodium carboxylates, namely, the monocarboxylate anion acetate, the dicarboxylate anion oxalate, and the tricarboxylate anion citrate, ordered in the sequence of increasing steric demand and efficiency for multidentate chelation of (partially) hydrated cations. Then, sulfate was paired with K^+^ to establish a comparison with its smaller homologue Na^+^. Finally, we combined sulfate with the divalent cation Mg^2+^ to test the hypothesis that the observed inhibiting effect of divalent chloride salts on nanofiber formation^26^ is not only due to the presence of chloride anions but also affected by the presence of divalent cations. Figure 4 shows the SEM images of the six salt-fibrinogen precipitates mentioned.

Fibrinogen films formed starting with 50 mM Na-oxalate (see Figure 4A) and 100 mM Na-acetate (Figure 4B), resulting in rough precipitates with fibrous features. For Na-acetate, the fibrinogen morphology showed some porosity, suggesting a possible coalescence of previously formed and precipitated fibers. On the other hand, films starting with 100 mM of Na-citrate (see Figure 4C) displayed localized fiber-like structures between elevated smooth, potentially coalescent domains, indicating citrate’s greater tendency to induce fibrinogen fiber assembly compared to acetate, as observed by Hense et al.^17^ Further investigations, e.g., applying sodium soaps,^53^ might reveal if the length of terminal alkyl chains in acetate-homologue monocarboxylates affects the shape of coprecipitated fibrinogen deposits. In contrast to the tested sodium salts of carboxylic acids, starting with 100 mM sodium sulfate induced fiber formation with a star-shaped fibrinogen morphology (see Figure 4D), similar to that reported for PBS.^20,21^ Previously, we showed that divalent salts form smooth films when the counterion was chloride.^26^ However, when we combined the divalent cation Mg^2+^ with sulfate at 100 mM concentration, a rough fibrinogen surface without nanofibrous regions was found (see Figure 4E). Finally, starting with 100 mM potassium sulfate also created rough fibrinogen structures (see Figure 4F), similar to those of 375 mM KCl. These results suggest that the monovalent cations Na^+^ and K^+^ and the divalent cation Mg^2+^, in combination with sulfate, lead to rough fibrinogen structures, differing from smooth films. Hence, we speculate that different cations influence differently the size and structure of fibrinogen-based precipitates. They might also affect the surface mobility of fibrinogen growth units attaching to the forming film, as seen in halite growth models and exemplarily expressed by a Damköhler number.^40^

Our SEM-based morphology analysis of dried fibrinogen precipitates shows the following pattern for sodium salts (c.f. Figures 2 and 4):

- NaPO_4_ and Na_2_SO_4_: full coverage with dense fibers;
- NaCl: fiber formation only below crystals;
- Na-citrate: fiber formation at localized spots surrounded by confluent regions;
- Na-acetate: rough regions with coarsely fibrous features;
- Na-oxalate: rough regions without defined fibers.

We conclude that SO_4_^2–^ anions lead to denser and more defined fiber networks than Cl^–^ anions as seen in Figure 2C for NaCl and Figure 4D for Na_2_SO_4_. Previously, Dumetz et al. showed that sulfate increases attractive interactions of proteins in concentrated salt solutions more than chloride, attributing this to water-mediated effects.^54^ Similarly, Metrick II and MacDonald showed that SO_4_^2–^ ions decrease protein solvation and thus increase protein stability, favoring salting-out effects (i.e., protein aggregation), while Cl^–^ ions enhance protein solvation and decrease protein stability in solution.^55^ These reports agree well with the trends in fiber assembly that we observed for fibrinogen in the presence of SO_4_^2–^ and Cl^–^.

Our findings suggest that not all polyvalent anions with sodium induce fiber formation. Neither Na^+^ or K^+^ cations nor SO_4_^–2^ anions alone can fully explain the formation or absence of fibrinogen nanofibers. Comparing Na_2_ SO_4_ to K_2_ SO_4_, we found that the latter results in a much flatter fibrinogen film, despite both cations sharing the same counteranion. This may be due to sodium’s higher affinity for protein surfaces^48^ and its more ordered hydration shell compared to potassium.^56^ This might indicate that sodium provides a more kosmotropic environment for fibrinogen than potassium and promotes nanofiber formation by altering the protein self-assembly process.

### 3.6 MD Simulations of Cation and Anion Interactions with the Fibrinogen D Domain

Both the new experiments presented in this paper and in our previous works^20,26^ have shown that fibrinogen fibers do not assemble upon drying in salt solutions with divalent cations, such as Mg^2+^; instead, they lead to the formation of smooth films. This suggests a fundamental difference in how mono- and divalent cations interact with the fibrinogen molecule. Furthermore, we observed that Na^+^ ions remained within the aggregates even after repeated washing with water, whereas K^+^ and divalent cations were removed entirely. We hypothesized that this difference arises from the different ways in which the ions form contacts with the protein due to their ionic radius and water shell structure. To further investigate these interactions, we simulated the Fg-D domain in the presence of different salts at pH 7. At this pH, the Fg-D domain carries a net total charge of −1e. Figure S11 in the Supporting Information presents the electrostatic potential map of the protein domain, highlighting the nonuniform charge distribution with potential adsorption sites for various anions and cations characterized by distinct positive and negative pockets, respectively. We simulated the protein at salt concentrations high enough to be representative of the onset of fiber formation during the drying process, which we estimate to be in the order of around 1 M (cf. Figure 1).

#### Interaction of Cations with the Fg-D Domain in the Presence of Chloride Anions

Figure 5 presents a comprehensive analysis of the interaction of different cations (Na^+^, K^+^, and Mg^2+^) with the Fg-D domain through three distinct approaches: distance distribution, ion retention within cutoffs, and hydration shell analysis, as mentioned in detail in Section 2.9.

**Figure 5:**
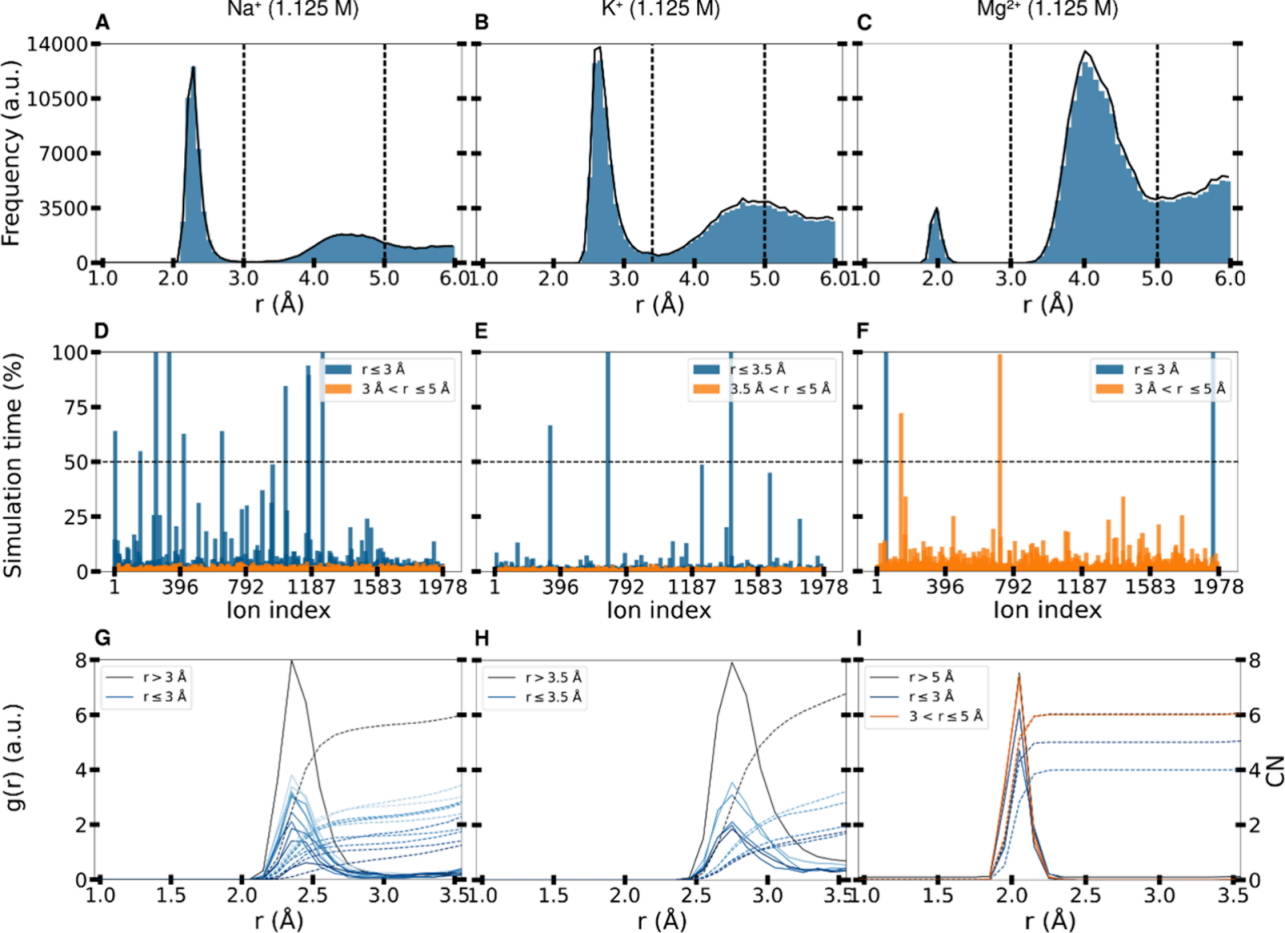
Simulation Analysis of Monovalent Cations (Na^+^, K^+^) and Divalent Cation (Mg^2+^). (A–C) Distance distribution analysis of Na^+^, K^+^, and Mg^2+^ cations from the Fg-D domain atoms (excluding hydrogen atoms). Each graph displays two distinct peaks, with the minima of these peaks used as cutoffs for further analysis. (D,F) Ion retention within cutoff: Percentage of residence time for each cation within the specified cutoff distance from the Fg-D domain. Na^+^ ions exhibit the highest residence time within the first cutoff, indicating a closer and more sustained interaction with the protein compared to K^+^ and Mg^2+^ ions. (G–I) Hydration shell of immobile cations. Radial distribution functions (*g*(*r*)) and coordination numbers (CN) of water oxygen atoms around the immobile cations (those within the cutoff distance and with a residence time exceeding 50%). The *g*(*r*) is shown as a continuous line, while CN is depicted with a dashed line. In the reference state (black line), cations not in contact with the protein maintain a hydration shell of up to six water molecules. Immobile Na^+^ and K^+^ ions within the first cutoff (blue gradients) show a significant loss of water molecules—up to four—indicating direct protein contact. In contrast, Mg^2+^ ions within the first cutoff retain most of their hydration shell, losing only one or two water molecules, suggesting a more loosely bound interaction with the protein. Mg^2+^ ions in the second cutoff (orange gradients) maintain their full hydration shell, indicating indirect contact with the protein via water molecules.

Figure 5A–C displays the distance-resolved distributions of cations around the Fg-D domain. The pronounced peaks indicate the most probable distances at which the ions are found near the protein domain. The closer peaks suggest a tight association of ions with protein residues. In contrast, more distant peaks might represent interactions mediated by adsorbed water molecules. For the monovalent cations (Na^+^ and K^+^), the first peak is more pronounced than the second, indicating a higher concentration of ions tightly bound to the Fg-D domain. However, for the divalent Mg^2+^ ion, the second peak is more pronounced, suggesting that a greater number of these ions interact indirectly with the protein via water molecules. Additionally, K^+^ ions tend to be slightly further away from the protein than Na^+^ ions, which is consistent with the different ionic radii (275 and 227 pm for K^+^ and Na^+^, respectively^26^).

Figure 5D,F quantifies the percentage of time each ion remains within defined cutoff distances from the protein, as determined by the minima of each peak in the distance distributions. Na^+^ ions remain within the first cutoff for a higher percentage of time compared to K^+^ and Mg^2+^ ions. In contrast, Mg^2+^ ions are more frequently found within the second cutoff distance, reflecting a preference for indirect interactions. We observed that some individual ions remain in contact with the Fg-D domain for >50% of the simulation time, which we refer to as immobile ions. Additionally, an analysis of the percentage of time the protein engages in contacts with any ion in solution reveals that the attractor sites on the protein vary in their ability to retain ions depending on their location in the protein surface (see Figure S12). Some residues can retain the same ion for a longer time, while others are less effective at retention. The diffusive movement of the mobile ions leads to continuous ion exchange at these latter sites. This restriction of ion movement by the protein is consistent with findings from simulations of the S6 ribosomal protein at different NaCl concentrations.^57^ The spatial distribution of immobile and mobile ions on the Fg-D domain is shown in Figure 6A–C. This figure also illustrates the higher charge screening provided by Na^+^ ions compared to other cations. Furthermore, the adsorption of these cations occurs predominantly at negatively charged pockets on the protein, as shown in Figure S11.

**Figure 6:**
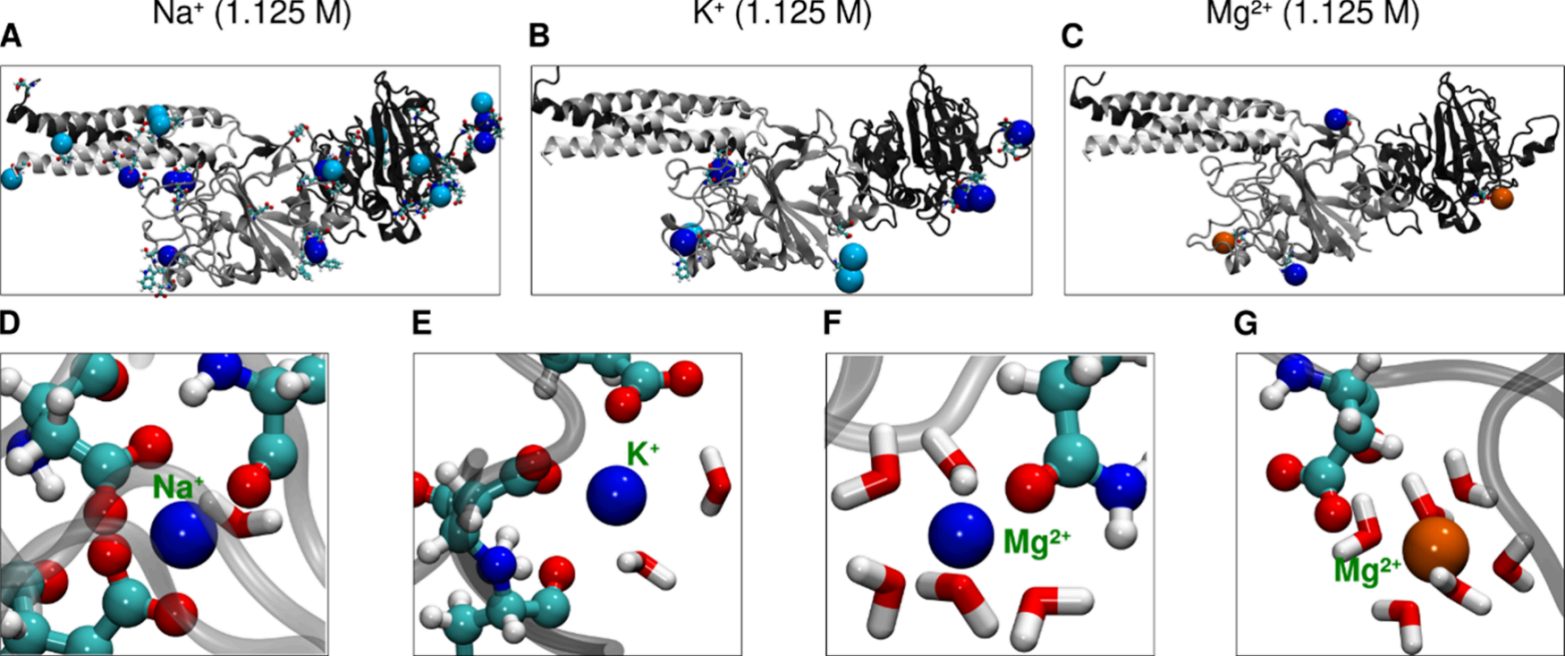
Representative snapshots of the immobile and mobile cations. (A–C) Both mobile (light blue) and immobile (dark blue) ions that are in contact with the protein are shown. Immobile ions are those that are within the first cutoff (direct contact) with the protein and that are within more than 50% of the residence time. Mobile ions are those where protein residues seize ions for more than 50% of the residence time, with ions exchanging. More Na^+^ ions are adsorbed to the protein compared to other cations. (D–F) Hydration shell around the contact ions. The trapping of cations by the protein’s oxygen atoms disrupts the water shell around the directly adsorbed ions. (G) An indirect contact of Mg^2+^ (shown in orange) with the protein. Here, Mg^2+^ has all six water molecules and is adsorbed to the protein via a water molecule.

The Na^+^, K^+^ and Mg^2+^ ions are coordinated by a shell of six water molecules,^26^ as shown by the coordination numbers of six for the three reference ions in bulk solution (black dashed lines in Figure 5G–I). However, the strength of the water–ion interaction decreases in the order Mg^2+^, Na^+^, and K^+^.^58^ Upon interaction with the Fg-D domain, the hydration shells of the ions change, as shown in Figure 5G–I. As can be seen, monovalent immobile cations exhibit disrupted hydration shells, remaining with fewer than two water molecules in close proximity. In contrast, even the few Mg^2+^ ions within 3 Å of the protein lose no more than two water molecules off their hydration shell compared with the reference. Instead, all ions lying within the second cutoff retain their hydration shell intact and undergo shell-mediated interactions with the protein. Figure 6D,E shows representative snapshots from the MD simulations that illustrate the trapping of Na^+^ and K^+^ ions, especially by carboxylate but also carbonyl groups of the protein. In particular, Figure 6F,G highlight the difference between direct and indirect interactions of Mg^2+^ with the Fg-D domain.

These findings support our previous hypothesis that monovalent cations, especially Na^+^, can form direct, long-lasting interactions with the protein by escaping from their water shells. The tight binding in protein binding pockets (see Figure 6D) likely explains why these ions are not entirely removed during washing. In contrast, Mg^2+^ maintains its water shell and is, therefore, more easily washed out. The differences between Na^+^ and K^+^ are more subtle but significant, with a much larger number of Na^+^ molecules becoming immobilized at the protein sites (c.f. Figure 5D,E), and at a closer distance (c.f. Figure 5A,B).

#### Interaction of Anions with the Fg-D Domain

A comprehensive analysis of the interaction of different anions (Cl^–^ and HPO_4_^2–^) and the influence of the divalent phosphate anion on the Na^+^ interaction with the Fg-D protein domain is presented in Figure 7. The distance distribution of Cl^–^ anions around the Fg-D domain (see Figure 7A) shows a first distinct peak and a second, more extended, accumulation region above about 3.5 Å. The HPO_4_^2–^ anion shows a single strong peak at a protein-phosphorus distance of approximately 3.6 Å. Notably, the O atoms of the phosphate anion are thus located much closer to the Fg-D domain than to the Cl^–^ anion. The distance distribution profile of Na^+^ in the presence of HPO_4_^2–^ closely resembles that of Na^+^ in the presence of Cl^–^, with a primary peak between 2 and 3 Å and a secondary peak between 3 and 5 Å.

**Figure 7:**
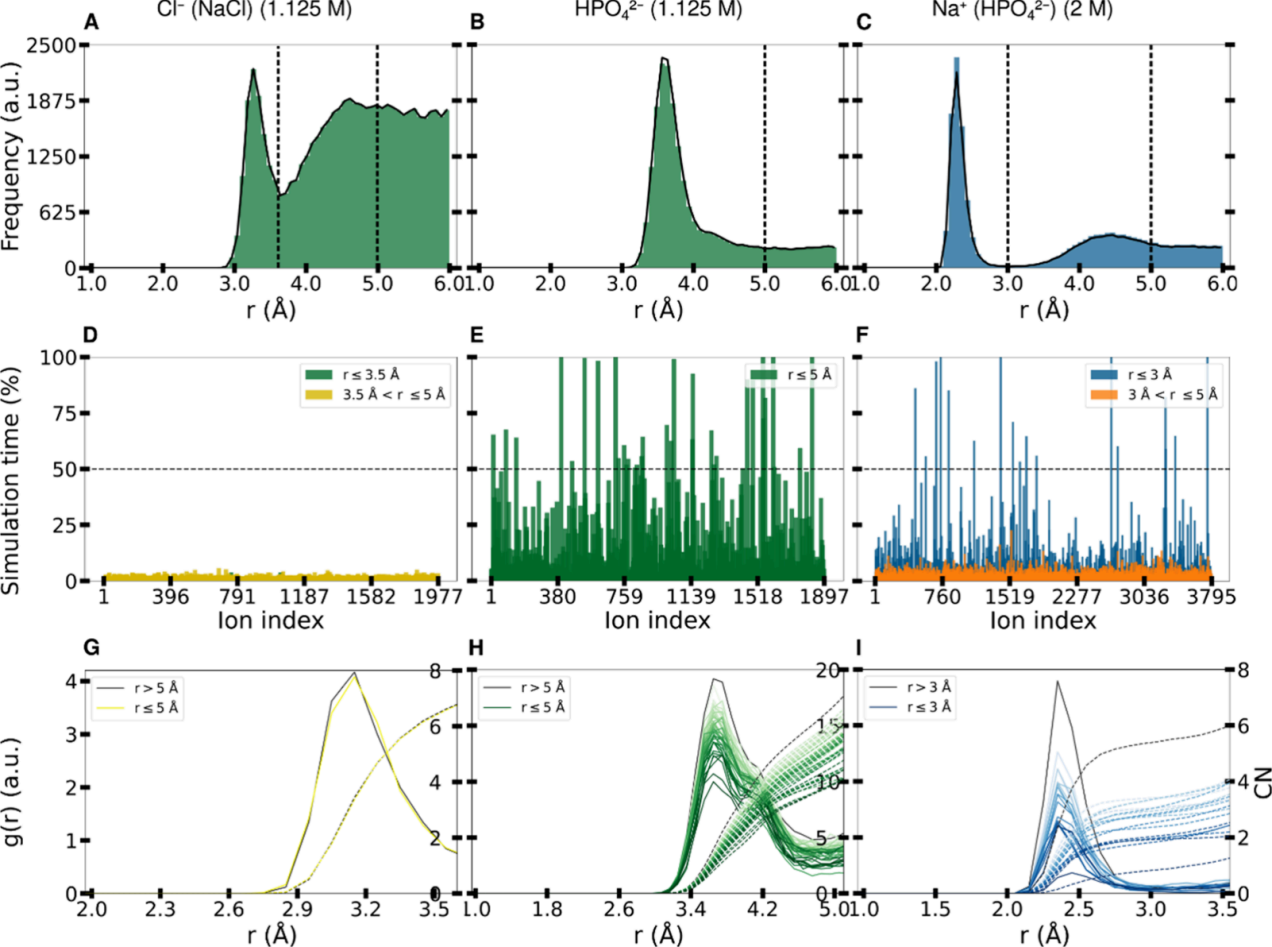
Simulation analysis of monovalent anion (Cl^–^), divalent phosphate anion (HPO_4_^2‑^), and the influence of divalent anions on cation adsorption (Na^+^ (HPO_4_^2‑^)). (A–C) Distance distribution of various ions from atoms of the Fg-D domain (excluding hydrogen atoms). For the divalent phosphate anion, the distance is measured from the phosphorus atom. For Cl^–^ and Na^+^ (HPO_4_^2–^), two cutoffs are used, determined by the minima of the two peaks. For HPO_4_^2–^, only one cutoff is used at 5 Å. (D–F) Residence time (% of simulation time) of each ion in contact with the Fg-D domain within the specified cutoff. Cl^–^ ions exhibit rapid diffusion with minimal residence time, predominantly within the second cutoff, indicating indirect ion attachment. Conversely, HPO_4_^2–^ and Na^+^ (HPO_4_^2–^) show a lot of ions with more than 50% of residence time closer to the protein. (G–I) Radial distribution functions (*g*(*r*)) and coordination number (CN) of water oxygen atoms around the adsorbed ions (ions within the protein cutoff with residence times exceeding 50%). *g*(*r*) is represented by a continuous line and CN by a dashed line. Similarly to Figure 5, both the HPO_4_^2–^ and Na^+^ (in phosphate) affect the hydration shell, indicating direct contact with the protein. In contrast, the water shell around the Cl^–^ ions is tightly bound with all six water molecules.

Figure 7D,F shows that HPO_4_^2–^ anions spend a much higher percentage of time in direct contact with the protein compared to Cl^–^ anions. Cl^–^ anions both below and above the 3.5 Å cutoff distance have a contact time of less than 5%, suggesting that the locally positive sites of the protein surface are unable to prevent the diffusion of Cl^–^ ions back into the solution.

The ion retention profile of Na^+^ in the presence of HPO_4_^2–^ resembles that of Na^+^ in the presence of Cl^–^, with Na^+^ ions spending a longer simulation time within the first cutoff near the protein. Figure 8A–C shows representative snapshots of the distribution of mobile and immobile ions. Since there are no immobile Cl^–^ ions (defined as those present for more than 50% of the simulation time), only mobile ions are shown. Compared with the Cl^–^ anion, a greater number of phosphate anions are found closer to the protein. Figure 8C–F illustrates how both the cation (Na^+^) and the anion (HPO_4_^2–^) are able to adsorb together at neighboring sites of the protein surface.

**Figure 8:**
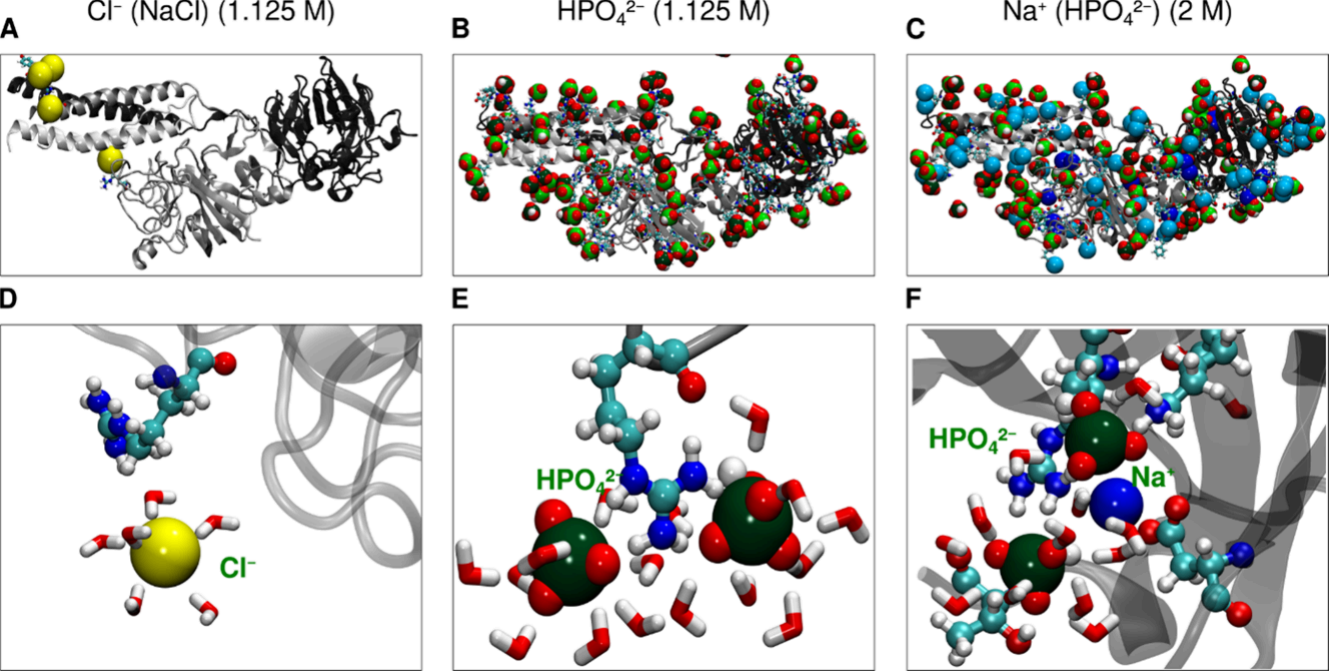
Representative snapshots of the mobile and immobile ions. (A–C) Both mobile (Cl^–^: yellow, HPO_4_^2–^: light green, Na^+^: light blue) and immobile ions (HPO_4_^2–^: dark green, Na^+^: dark blue) that are in contact with the protein are shown. More HPO_4_^2–^ anions are adsorbed to the protein in comparison with the Cl^–^ anion. More Na^+^ ions are adsorbed in the presence of HPO_4_^2–^ anion. (D–F) Hydration shell around the contact ions. The tightly bound water shell (with all six water molecules) of the Cl^–^ ion is shown. The trapping of HPO_4_^2–^ by the protein’s atoms disrupts the water shell around the directly adsorbed ions. The trapping of the Na^+^ ion by the phosphate anion and the protein atoms is also seen.

As a consequence of the adsorption, the hydration shell of immobile HPO_4_^2–^ anions is disrupted (see Figure 7H,E), whereas all Cl^–^ ions preserve an intact hydration shell with six water molecules (see Figure 7G,D). Figure 8D,E further illustrates the interaction of Cl^–^ and HPO_4_^2–^ anions with the protein’s atoms with a full or incomplete hydration shell. Figure 8F highlights the sandwiching of the Na^+^ ion between protein oxygen atoms and the oxygen atoms of the divalent phosphate anion with a disrupted hydration shell.

We also analyzed the interaction of the monovalent phosphate anion H_2_PO_4_^–^ with the Fg-D domain (see Supporting Information, Figures S13A,C,E and S14A,C). Similar to the divalent phosphate, a large number of monovalent phosphate anions are found close to the protein (3–5 Å), where they spend a large percentage of the simulation time. The trapping of Na^+^ ions by the monovalent phosphate anion is shown in Figure S14B,D). These findings indicate that in the presence of phosphate salts, both the cation and anion contribute to the charge screening of the protein, a phenomenon not observed with chloride salts. In the presence of chloride salts, only the cations are adsorbed onto the protein. The direct adsorption of chloride ions is observed only at very high concentrations (2 M) and is still limited to mobile ions, as shown in the Supporting Information, Figure S15. In our recent review on fibrinogen fiber assembly *in vitro*,^1^ it was determined that the number of uncompensated electrokinetic charges changes with increasing ionic strength of the solution, approaching an equilibrium value of zero. This change in charge can be correlated with increased electrical screening of ions at higher concentrations, as observed in our study.

It is important to note that our MD simulations focused on the fibrinogen D domain and revealed how specific ions, particularly kosmotropic phosphate species, preferentially bind and modulate local protein–protein interactions to promote aggregation. However, in our experiments showing fiber assembly in the presence of monovalent ions, a full-length fibrinogen molecule was used, which contains additional domains that can also influence fiber formation. Consequently, the hierarchical organization of aggregated fibrinogen into ordered nanofibers likely depends on interactions beyond the D domain, such as ion-mediated electrostatic shielding. While our current MD simulations already provide valuable insights into the initial ion-fibrinogen interactions, further simulations encompassing other fibrinogen regions will be helpful to fully unravel the mechanisms linking ion binding at the molecular level to supramolecular fibrinogen fiber assembly.

### 3.7 Kosmotropic Ion Pairs Drive Fiber Assembly From Highly Saline Fibrinogen Solutions

In our recent review, we showed that ions play a more significant role than the substrate surface properties during *in vitro* fiber assembly of fibrinogen.^1^ Although many studies previously included metal ions during different fiber assembly processes, their effect on fiber formation remained unclear, and no classification of fibrinogen solidification based on the Hofmeister series has yet been presented.^20,21,25,59–68^ In the present work, we have found that the sole consideration of different cations or different anions, as they would occur in a 1D Hofmeister series, is not sufficient to explain the various fiber morphologies obtained during drying-driven fibrillogenesis. Rather, both these findings and our earlier work on the influence of divalent ions on fibrinogen precipitation^26^ can be rationalized in terms of a two-dimensional Hofmeister-like series that summarizes the tendency of fibrinogen to form fibers based on the combination of anions and cations present during drying (see Figure 9). Ordinate and abcissa order anions and cations by their kosmotropic and chaotropic effects, respectively. Exemplary SEM images included in the 2D-Hofmeister series illustrate the impact of different anion–cation pairs on forming distinct patterns consisting of more or less thin or coalesced fibers or smooth films.

**Figure 9:**
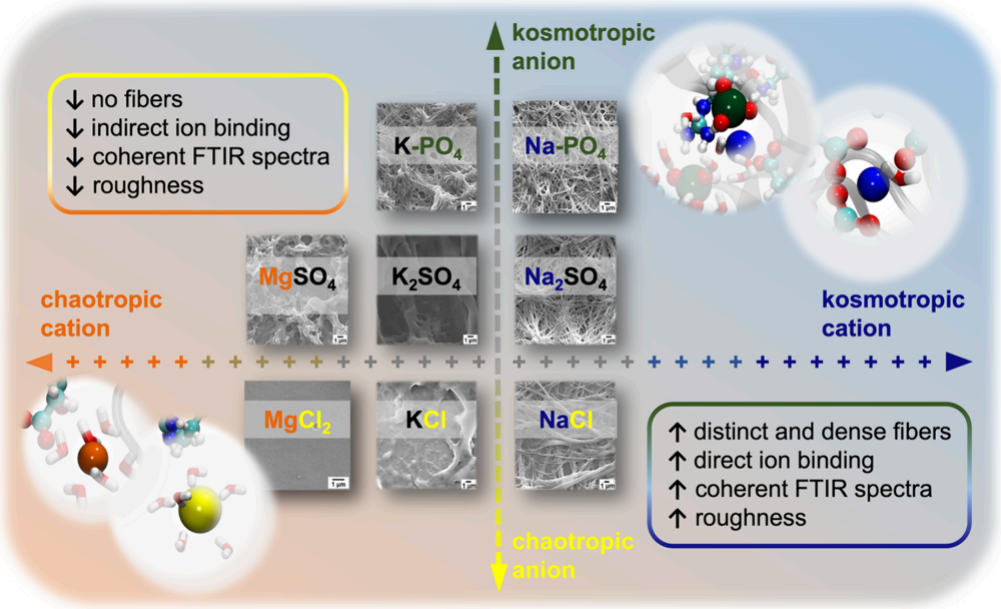
Two-dimensional Hofmeister series describing the influence of different ions on the morphology of fibrinogen precipitates. The abscissa ranges from chaotropic cations on the left to kosmotropic cations on the right, and the ordinate ranges from chaotropic anions at the bottom to kosmotropic anions on the top. The combination of a kosmotropic cation with a kosmotropic anion (e.g., Na-PO_4_) leads to very dense fiber networks as shown in the green shaded quadrant at the top right, whereas a chaotropic cation combined with a chaotropic anion (e.g., MgCl_2_) yields a very smooth structure as indicated by red shading at the bottom left. An intermediate state with the combination of a kosmotropic cation with a chaotropic anion (e.g., KCl) leads to a rough surface with no clear fiber formation. Image of fibrinogen dried in the presence of MgCl_2_ is modified and reproduced with permission from ref 26. Copyright 2021, American Chemical Society.

Kosmotropic ions are known to stabilize and salt out proteins, while chaotropic ions have an opposite salting-in effect.^69^ Here, we highlight that a combination of kosmotropic cations and anions is associated with a higher capacity to assemble fibrinogen fibers. Sulfate and especially (protonated) phosphate lead to thinner fibers and rougher surfaces than chlorides.^70^ In our study, this is reflected by the higher molar concentrations of alkali chlorides needed to induce fibrillogenesis compared to those of Na_2_HPO_4_ and K_2_HPO_4_ (see Figure 1). Moreover, monovalent kosmotropic cations like Na^+^ can form fibers with slightly kosmotropic anions like Cl^–^, while monovalent chaotropic cations like K^+^ only form fibers with strongly kosmotropic anions like HPO_4_^2–^. Divalent salts do not form fibers regardless of the anion, even if highly kosmotropic (e.g., SO_4_^2–^). These results underline the importance of both cations and anions in fibrinogen fiber formation, countering previous studies on protein aggregation or assembly upon drying that suggested anions alone^17,69,71,72^ or cations alone^25^ are more relevant.

Our results complement those of Hense et al.,^17^ who studied the influence of Hofmeister salts on fibrinogen fiber formation by varying anions with Na^+^ cations but did not study highly saline conditions where salting-out effects may dominate. This may explain why they observed fewer, thinner fibers with chaotropic anions such as acetate, whereas we found thicker, coalesced fibers with Cl^–^ due to a ripening effect. In contrast to our previous study,^26^ Hense et al. also showed fibrinogen fiber formation at 5 °C with CaCl_2_ and MgSO_4_ without external triggers (e.g., drying) allowing long aggregation times.^73,74^ They found more defined fibers using Na_2_SO_4_ with MgCl_2_ ^74^ and observed spontaneous protofibril formation at 4 °C for fibrin-rich fibrinogen solutions with low sodium phosphate concentration.^18,19^ Conversely, at 37 °C fiber assembly required NaCl addition to CaCl_2_,^71^ indicating two regimes of salt-induced fibrinogen fiber assembly: slow, low-salt assembly at low temperatures and faster assembly with high monovalent salt and fibrinogen concentrations.

Many authors have studied how Hofmeister salts influence different types of self-assembly systems, proposing various explanations,^69,71,72,75^ but recent evidence shows that this complex phenomenon cannot be explained by a single theory.^69,72^ Our MD simulations show that the hydration properties of both cations and anions and their types of interaction with fibrinogen differ greatly depending on the ionic species. In particular, Cl^–^ ions behave as a highly diffusive cloud with very little residence time for the ions at protein adsorption sites. In contrast, partly protonated phosphate ions form stable direct contacts with the protein surface, partly also paired with especially Na^+^ cations. The latter appear to bind in large amounts and form tight bonds with carboxyl and carbonyl functional groups of the protein. In contrast, Mg^2+^ ions interact almost entirely in an indirect way, without leaving their own hydration shell, and even when they do, the residence time at the protein surface is very low. Thus, the fiber formation capability of kosmotropic ion pairs appears to correlate with their ability to bind stably (i.e., with long residence time) and frequently (i.e., in large amounts) to the protein.

These findings are consistent with the fact that neither Cl^–^ nor divalent cations Mg^2+^ were detected in our present and past XPS or EDX studies.^26,76^ In the present work, we show that repeated washing allows for removing K^+^ entirely and Na^+^ in part, consistent with its higher binding affinity observed in the simulations.^48^ Importantly, the combined presence of phosphate leads to a further stabilization of bound ions, once again stressing the role of ion pairs rather than single ionic species.^72^

Considering all these aspects and our recent results on the targeted fibrinogen fiber formation driven by drying highly saline aqueous formulations, we believe that sodium phosphate provides the best ion/counterion combination for fibrinogen fiber assembly, as it favors all possible interactions of the water-fibrinogen-salt triad in a synergic way. Beyond the mechanistic understanding of the ion-driven fibrinogen fiber assembly, our findings have direct relevance for various biomedical applications. Since the resulting nanofiber scaffolds can mimic native blood clot and extracellular matrix features, they hold great promise for wound healing and skin regeneration or as scaffolds for tissue engineering.

## 4 Conclusions

By combining fibrinogen with four different salts with monovalent cations (NaCl, KCl, Na_2_HPO_4_, and K_2_HPO_4_), SEM imaging and Aq mapping analysis revealed a coprecipitation of salts and fibrinogen with varying morphologies. These range from smooth (KCl), rough, and locally fibrous (NaCl), to highly and ubiquitously rough/fibrous (Na-PO_4_ and K-PO_4_) topography, with the latter forming the densest network with the smallest fiber diameters. FTIR analysis of fibrinogen precipitates showed that conformational changes are not clearly correlated with fiber assembly. XPS analysis revealed that ion retention in fibrinogen precipitates after repeated washing steps is detectable only for samples with clear fiber formation and more persistent for Na^+^ than for K^+^. Divalent cations were rinsed out of the fibrinogen precipitates upon washing, consistently with the trends in adsorption residence time and tightness of protein-ion association revealed by MD simulations. Fibrinogen fibrillogenesis was more pronounced for polyvalent phosphate anions (HPO_4_^2–^) than for chloride anions, which strikingly correlates with the much tighter and more persistent adsorption of the former anions in the MD simulations. In general, pairs of kosmotropic ions and cations (e.g., Na^+^ and SO_4_^2–^ or HPO_4_^2–^) led to the formation of well-defined, thin fibers, whereas pairs of chaotropic ions (e.g., Mg^2+^ or K^+^ and Cl^–^) yielded smooth to mildly rough precipitates without fibers. In mixed kosmotropic/chaotropic pairs, the anionic species tend to dominate the fiber formation capability during drying, although not entirely (coarse fibers were formed in the presence of Na^+^ and Cl^–^, for example). The absolute ionic strength also plays a role, with higher starting salt concentrations promoting fibrillogenesis. Our findings can be rationalized by means of a here-proposed two-dimensional Hofmeister series, which may serve as a guide for the optimization of drying-based process routes toward fibrous fibrinogen scaffolds for regenerative medicine.

## Supplementary Material



## Notes

Additional experimental data, including SEM and light microscopy images, Aq profiles and maps, fiber diameter and surface roughness analyses, and supplementary results from molecular dynamics simulations (PDF)
