# Supplementary material for: Hofmeister-Driven Ion Pairing in Monovalent Salts Directs Fibrinogen Nanofiber Assembly during Drying
Source: Biomacromolecules. 2025 Sep 5;26(10):6755–72. doi: 10.1021/acs.biomac.5c01056 (PMC12522127; doi:10.1021/acs.biomac.5c01056)
Supplement: Supplementary file 1 [file bm5c01056_si_001.pdf]

Supporting information

Hofmeister-driven ion pairing in monovalent salts directs fibrinogen nanofiber assembly during drying

Stephani Stamboroski[1,2], Aparna Sai Malisetty[3,6], Kwasi Boateng[1,2], Jana Lierath[1,2], Jonas Aniol[1], Peter Schiffels[1], Paul-Ludwig Michael Noeske[1,4], Lucio Colombi Ciacchi[3,6], Susan Köppen[3,6], Dorothea Brüggemann[5,6,]*

[1] Fraunhofer Institute for Manufacturing Technology and Advanced Materials IFAM, Wiener Strasse 12, 28359 Bremen, Germany

[2] Institute for Biophysics, University of Bremen, Otto-Hahn-Allee 1, 28359 Bremen, Germany

[3] Hybrid Materials Interfaces Group, Faculty of Production Engineering and Bremen Center for Computational Materials Science, University of Bremen, 28359 Bremen, Germany

[4] University of Applied Sciences Bremerhaven, An der Karlstadt 8, Bremerhaven 27568, Germany

[5] City University of Applied Sciences, Neustadtswall 30, 28199 Bremen, Germany

[6] MAPEX Center for Materials and Processes, University of Bremen, 28359 Bremen, Germany

Table S1:

Table S1: Details of fiber diameter analysis from representative SEM images. Fibrinogen fibers were prepared by drying mixtures of 2.5 mg/mL fibrinogen with (A) 375 mM NaCl, (B) 2.5x PBS, (C) 100 mM Na-PO_4 and (D) 100 mM K-PO_4. Average fiber diameter measurements were conducted by measuring 50 randomized positions per SEM image (indicated by red lines) with the ImageJ Software. The thinnest average fiber diameters were obtained with Na-PO_4, followed by K-PO_4, PBS and NaCl. For 375 mM KCl no fiber diameters were obtained because no fibers had formed during fibrinogen precipitation.

<table>
<tr>
<td>(A) 375 mM NaCl: 371 ± 131 nm
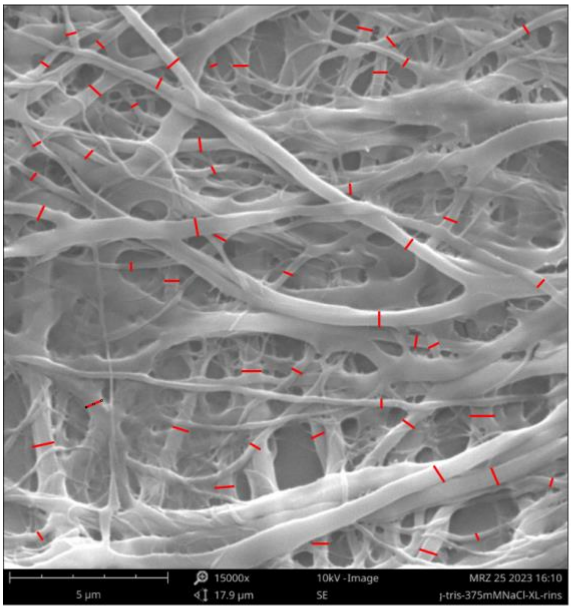
</td>
<td>(B) 2.5x PBS: 301 ± 76 nm
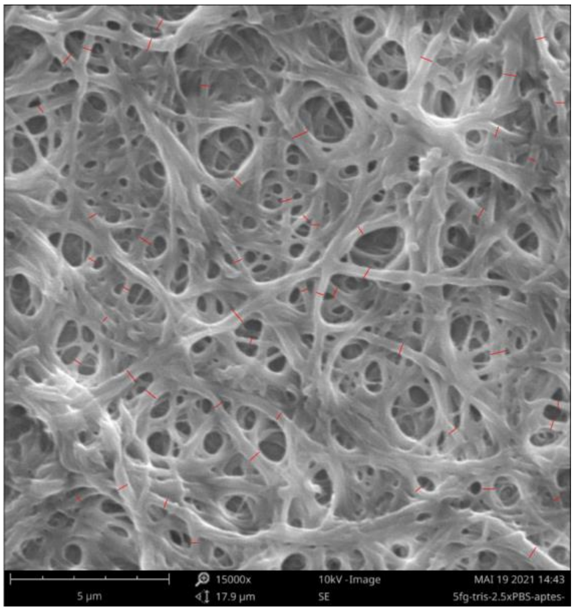
</td>
</tr>
<tr>
<td>(C) 100 mM Na-PO_4: 228 ± 49 nm
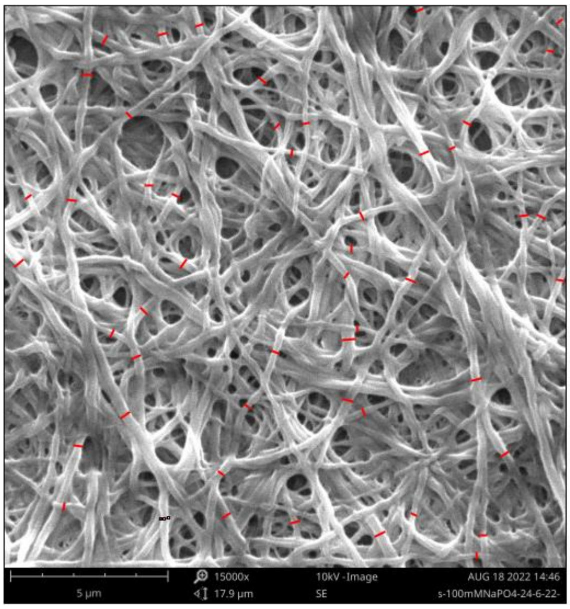
</td>
<td>(D) 100 mM K-PO_4: 233 ± 67 nm
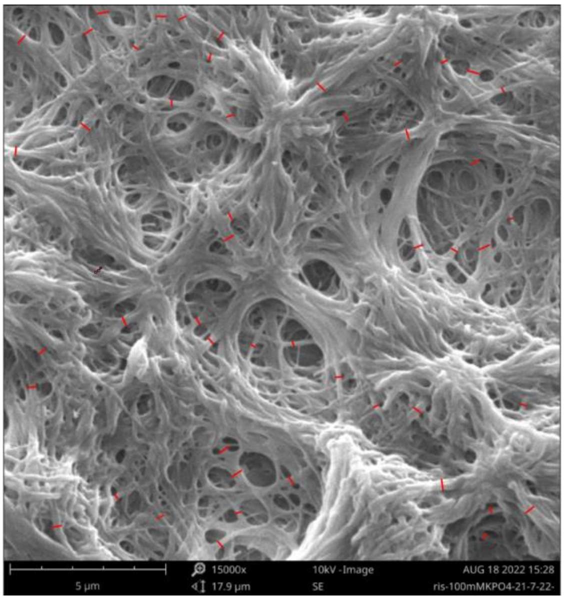
</td>
</tr>
</table>

Figure S1:

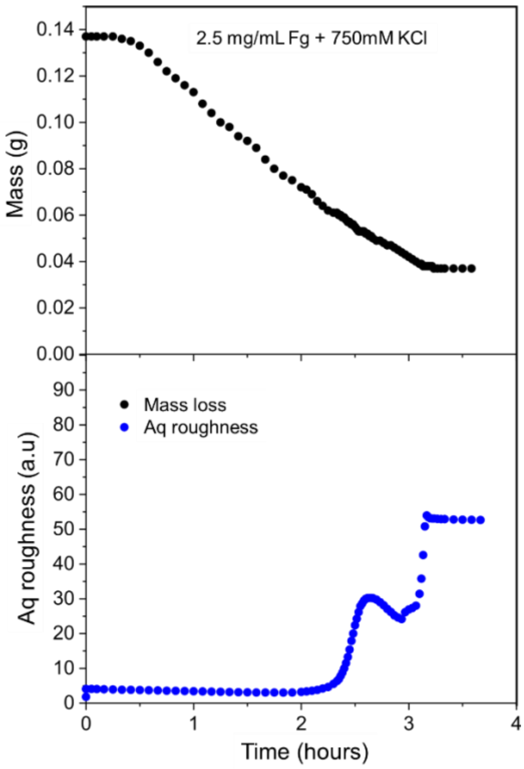

Figure S1: Mass loss correlation with Aq roughness during fibrinogen drying. Example of the linear mass decrease during drying (water evaporation) and *in situ* measurement of the Aq roughness as a function of time of one droplet containing a mixture of 2.5 mg/mL fibrinogen (Fg) and 750 mM KCl applied on the surface of a gold substrate. The first increase in Aq roughness happened when 75-80% of the water had evaporated, and the last increase was observed when most of the water had evaporated (95%)

Figure S2:

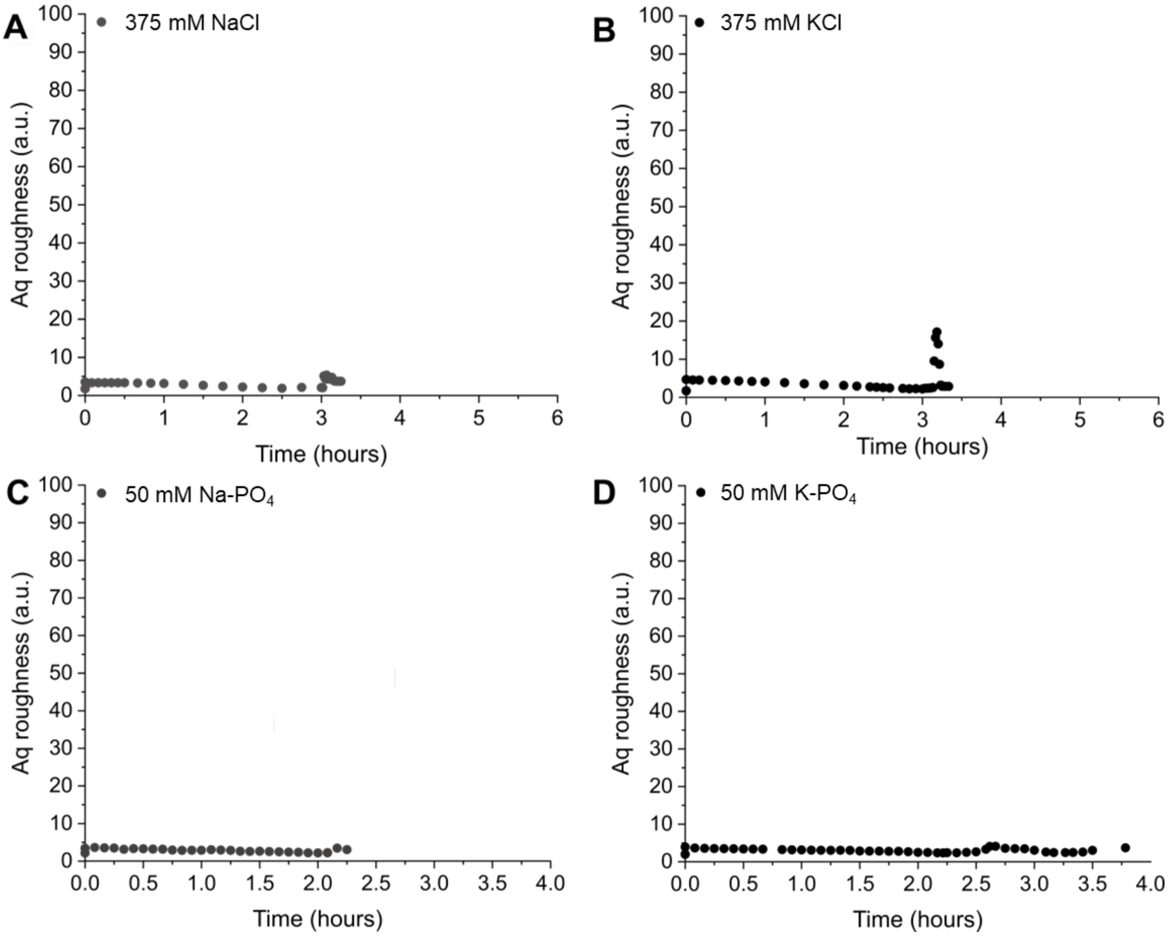

Figure S2: Aq curve profiles from light scattering analysis of monovalent salt precipitation on gold. In each plot, the round dots represent the change in Aq surface roughness during the drying of droplets containing (A) 375 mM NaCl (in Tris), (B) 375 mM KCl (in Tris), (C) 50 mM Na-PO_4, and (D) 50 mM K-PO_4. The initial Aq values at 0 h represent the roughness of the underlying gold substrate before the salt solutions were added. For all tested salts, no significant change in the Aq roughness was observed during drying. The final Aq roughness values around 1-2 indicate very smooth surfaces. When compared to Figure S3, we can infer that at the center of the samples no bigger salt crystals were formed and therefore no high Aq roughness was recorded.

Figure S3:

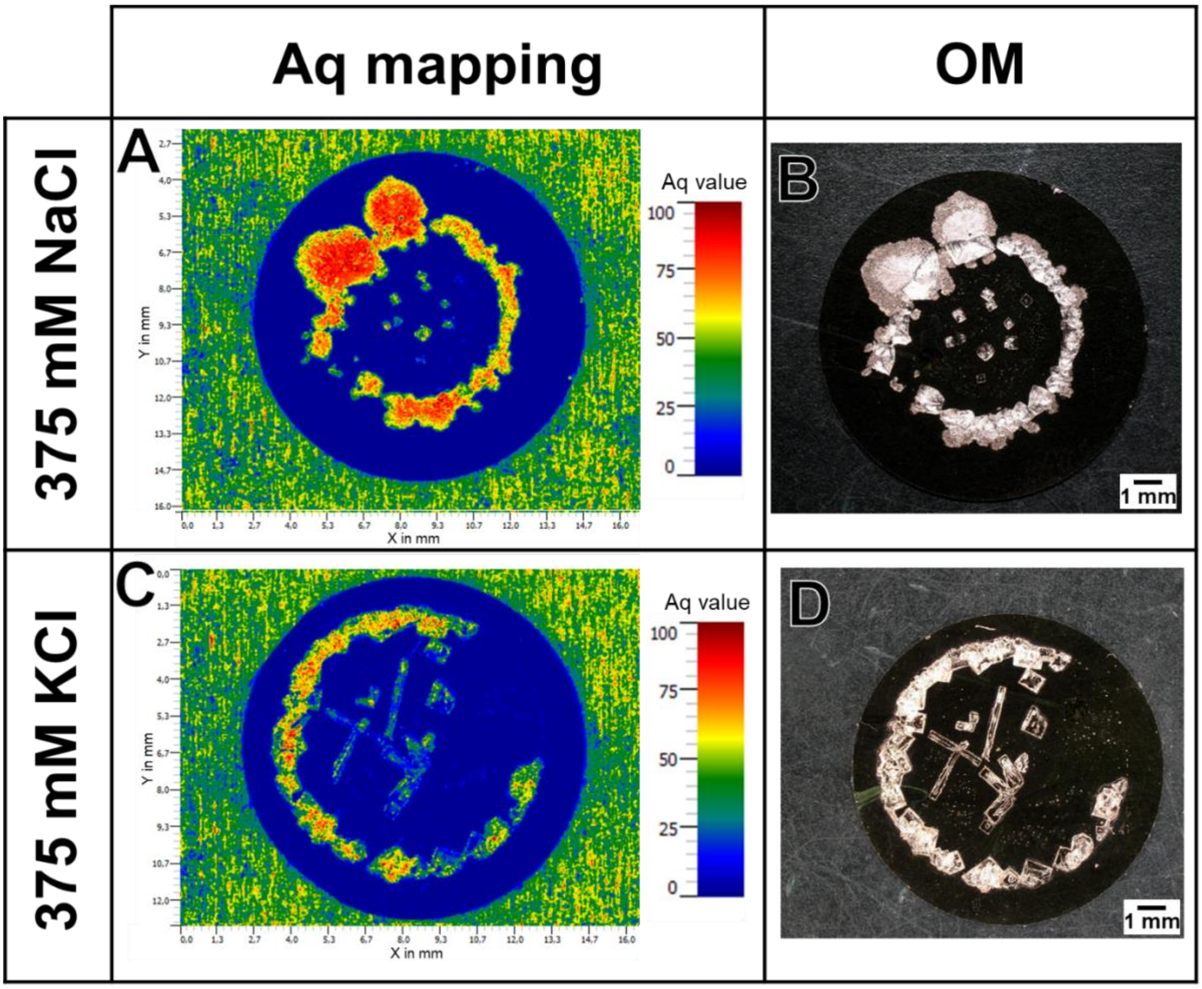

Figure S3: Surface features of dried salt crystals on gold substrates. (A) Aq based survey images and (B) optical light microscopy (OM) images of 375 mM NaCl, (C) Aq based survey images and (B) OM images of 375 mM KCl. For both salts, light microscopy images correspond to Aq-mapping with red color being an indication of rougher and more elevated areas. Salt crystals precipitated at the outer edges of the droplet, i.e., at the pinning line while the center was free of bigger salt crystals, revealing a very low Aq value at the center of both samples, corresponding well with the data obtained for *in situ* analysis of drying of salts alone without fibrinogen as presented in **Figure S2**.

Figure S4:

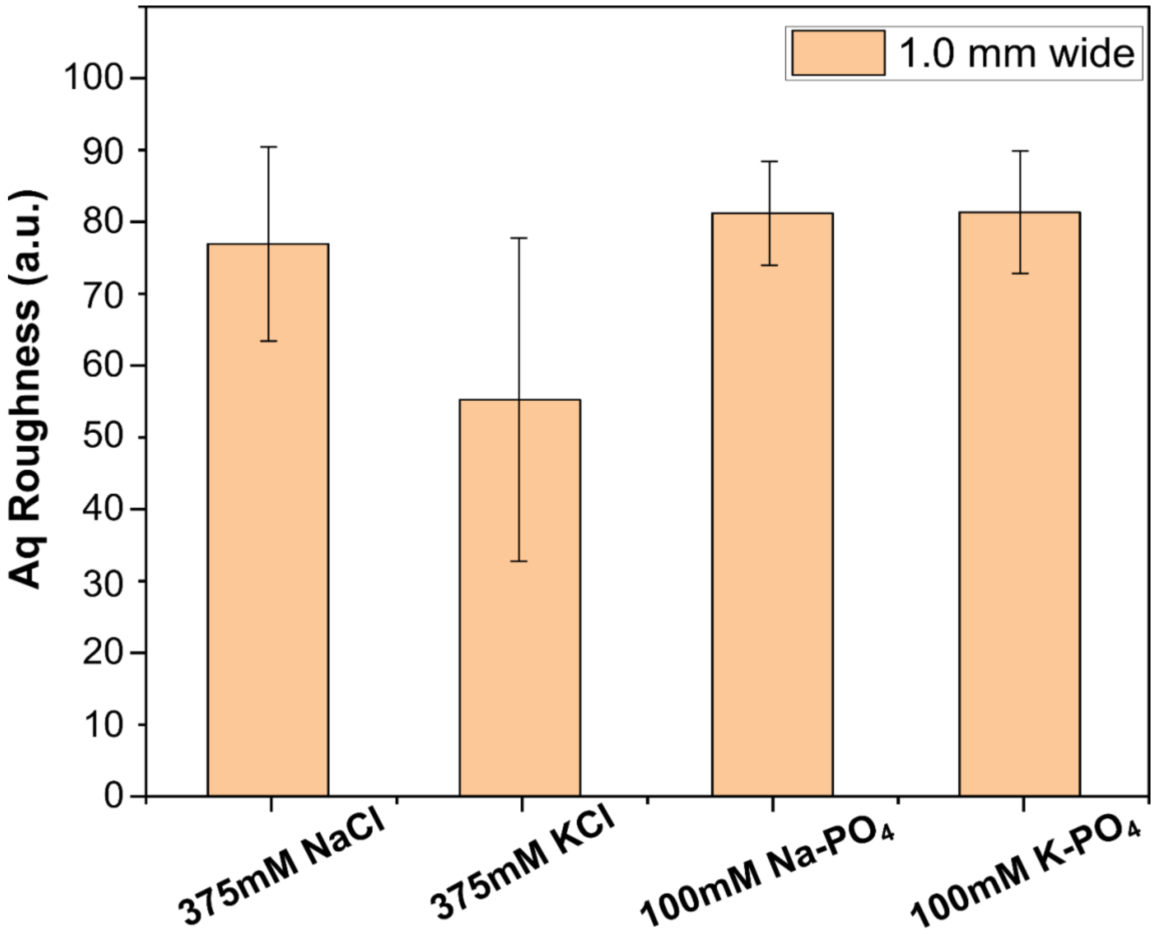

Figure S4: Average Aq roughness for 2.5 mg/mL fibrinogen dried in the presence of different monovalent salts after cross-linking and washing. The average Aq roughness was recorded in a 1 mm wide central regions of the samples presented in Figure 2. A smooth surface morphology (375 mM KCl, no fibers) led to the lowest Aq value, low density fiber (375 mM NaCl) yielded the second lowest Aq values, whereas more dense fibers (Na-PO_4 and K-PO_4) were associated with the highest Aq values and the lowest standard deviations.

Figure S5:

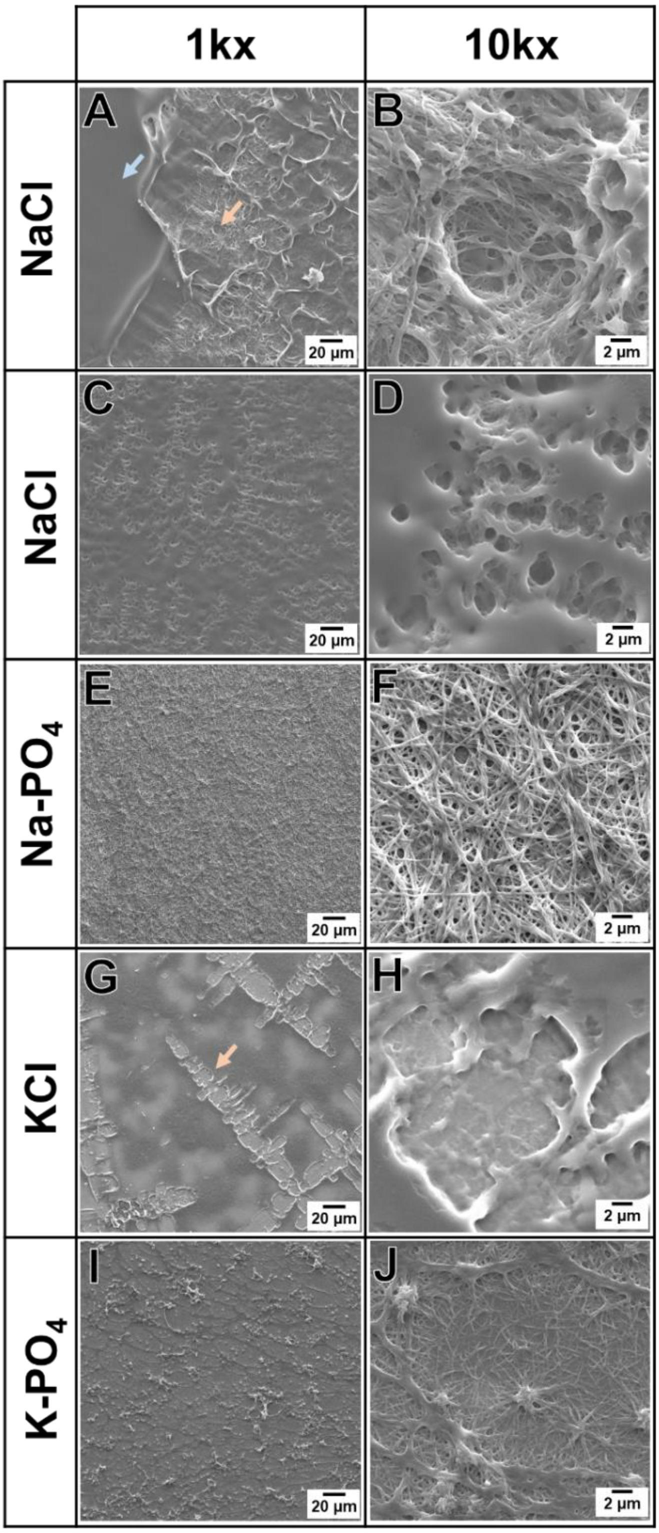

Figure S5: SEM-based local detail images of fibrinogen samples after drying, crosslinking and washing mixtures of 2.5 mg/mL fibrinogen and different salts. (A, B, C, D) 375 mM NaCl, (E, F) 100 mM Na-PO_4, (G, H) 375 mM KCl and (I, J) 100 mM K-PO_4. Image on the left show bigger regions of the samples at 1 kx magnification, while images on the right side show detailed parts at 10 kx magnification of a zoomed area of the same region. For NaCl, two different regions are presented to show the different morphologies observed along the same sample. The blue arrow in A indicates the elevated smooth region and the orange one shows the fibrous region, which is then shown in detail in B. For Na-PO_4 and K-PO_4, the fibers were distributed evenly across the sample. For KCl, the orange arrow in G points to the zoomed region presented in H.

Figure S6:

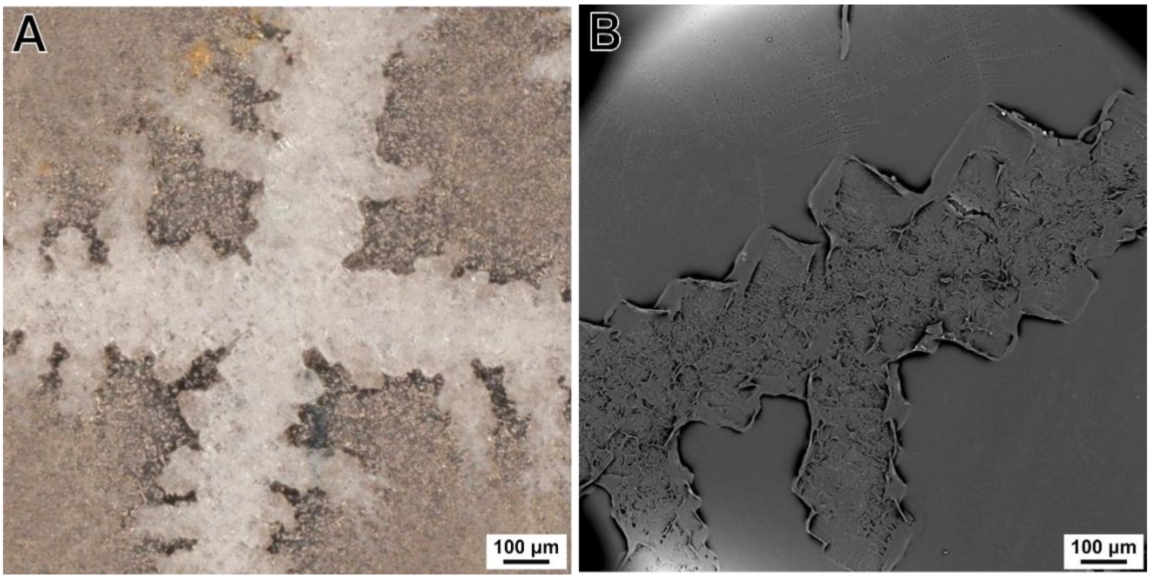

Figure S6: Fibrinogen precipitation in the presence of NaCl. (A) Optical microscopy image of 2.5 mg/mL fibrinogen with NaCl after drying and crosslinking (adapted from [1], licensed under a Creative Commons Attribution 4.0 International License), (B) Back scattered SEM image of 2.5 mg/mL fibrinogen with NaCl after drying, crosslinking and an additional rinsing step. The optical microcopy image in (A) shows the NaCl dendrites crystals formed during drying, and the SEM image in (B) shows the morphology of the fibrinogen layer below the NaCl dendrite crystals after washing, which preserved the fibrinogen nanofibers that had formed below the salt crystals. Morphological details of the fibrinogen sample shown in (B) are shown in **Figure 5**A-D.

Figure S7:

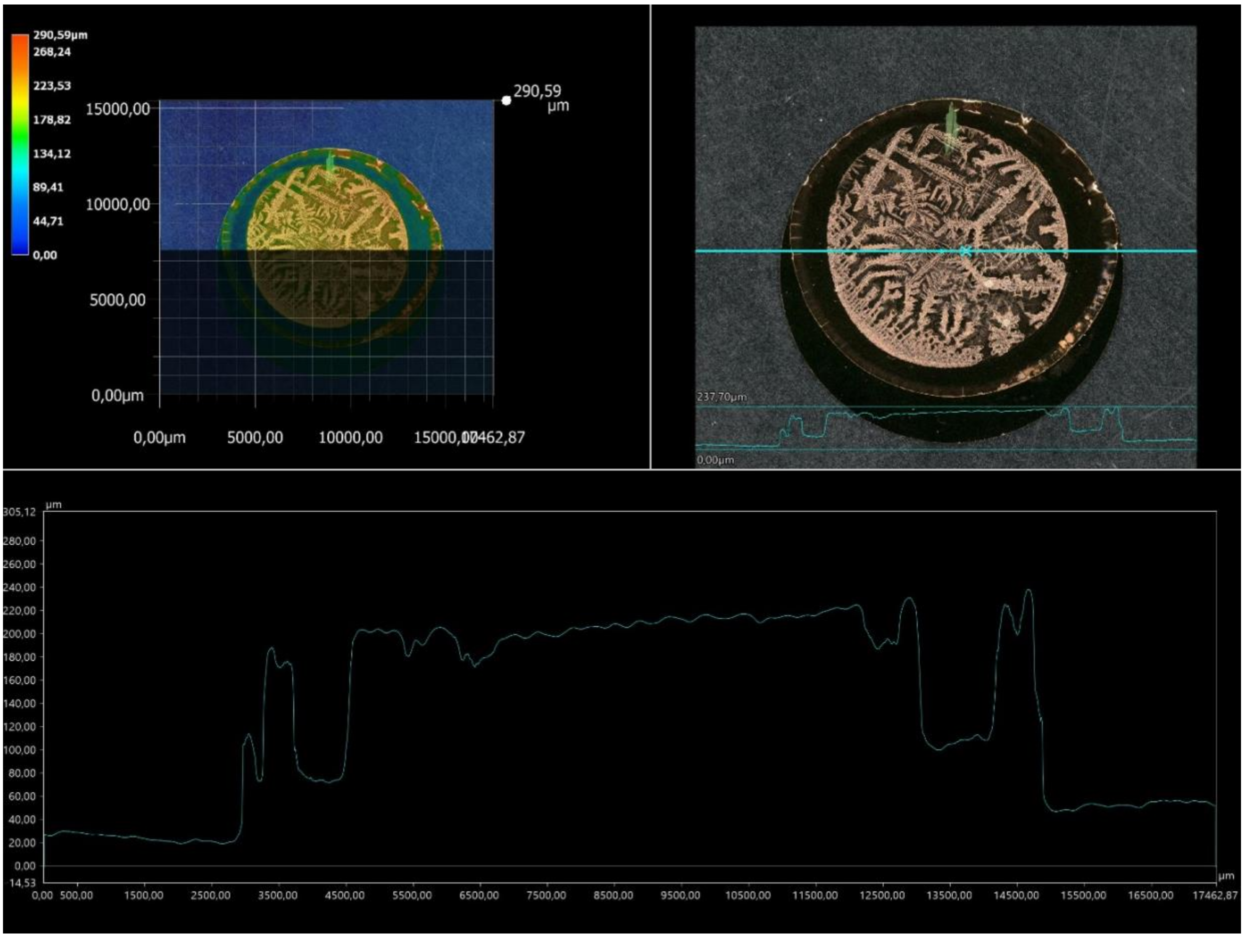

Figure S7: Surface topography of 2.5 mg/mL fibrinogen dried with 375 mM KCl in 5 mM Tris. Sample height profiles obtained from 3D optical microscopy after drying a mixture of 2.5 mg/mL fibrinogen and 375 mM KCl (in 10 mM Tris) showed how the thickness and salt crystal size varied along a representative horizontal line across the sample. The precipitated fibrinogen film has a thickness of at least a 100 µm.

Figure S8:

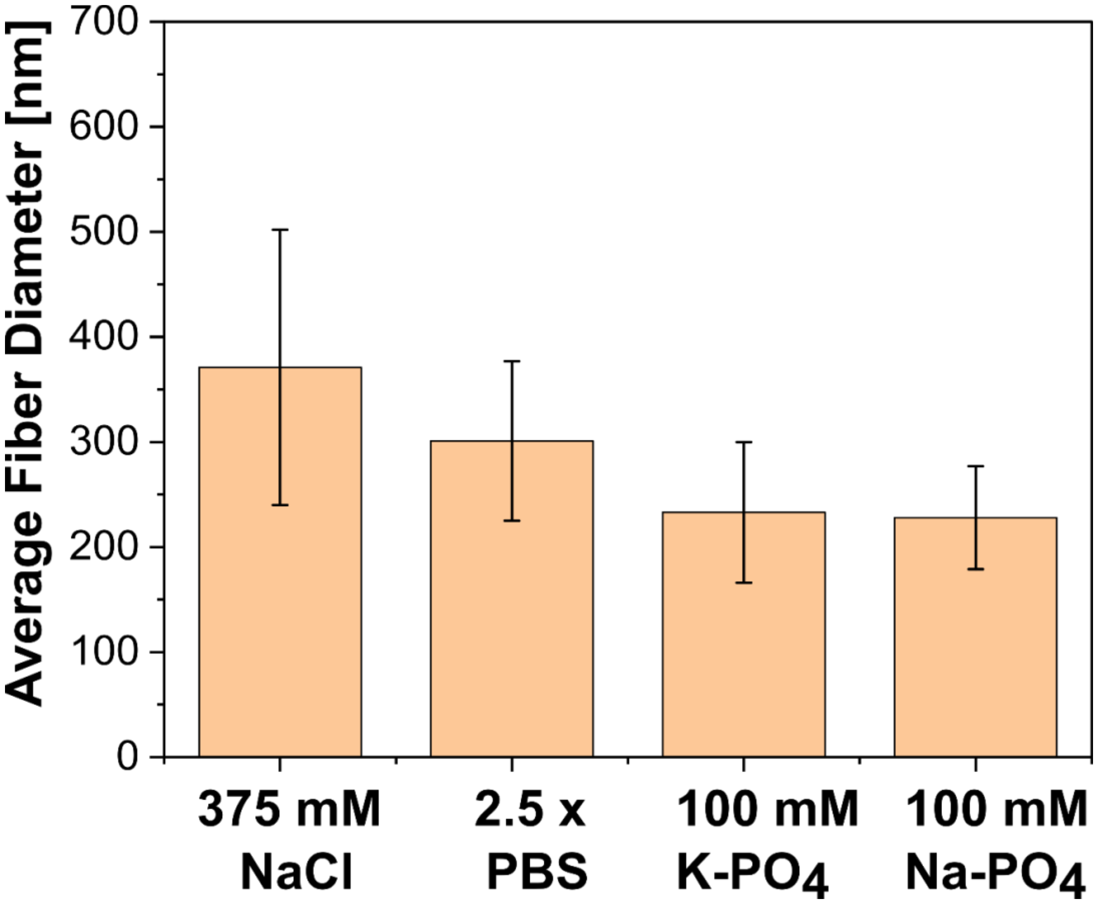

Figure S8: Average diameter of fibrinogen fibers assembled with varying monovalent salts. Average fiber diameters of samples prepared after drying 2.5 mg/mL fibrinogen in the presence of different salts were obtained from manual measurements with the Image J software at 50 distinct positions of SEM images (see **Table S1**). Fibrinogen precipitated from Na-PO_4 had the densest fiber network with the smallest average fiber diameter and lowest standard deviation, K-PO_4 resulted in the second densest fiber network with the second smallest fiber diameter, while PBS showed the third densest fiber network and second thicker fiber diameter, and NaCl yielded the least dense fiber network with the largest average fiber diameter and highest standard deviation.

Figure S9:

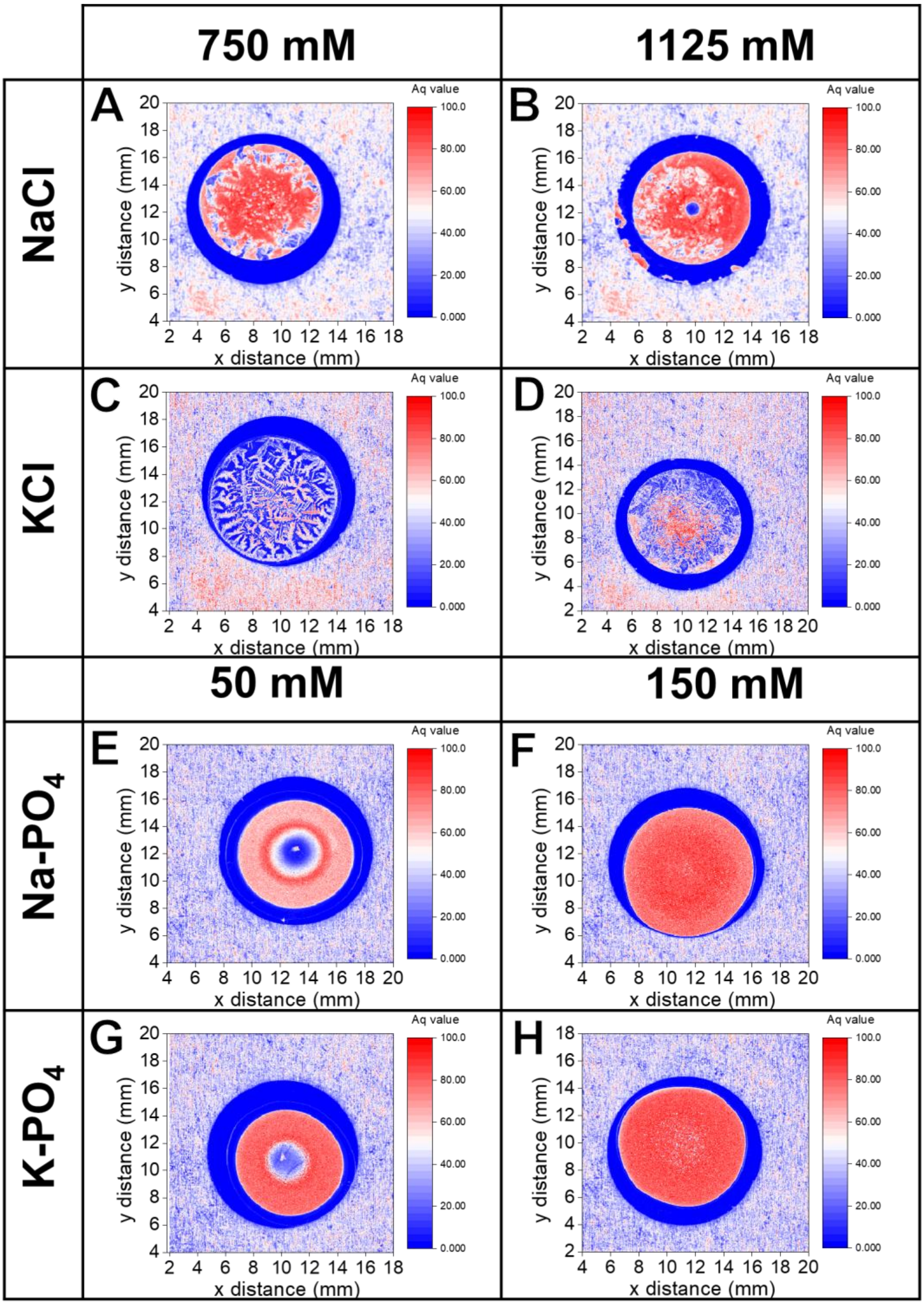

Figure S9: Influence of increased salt concentration on the surface roughness. Aq-based survey images of samples prepared after drying 2.5 mg/mL fibrinogen in the presence of (A) 750 mM and (B) 1125 mM NaCl, (C) 750 mM and (D) 1125 mM KCl, (E) 50 mM and (F) 150 mM Na-PO_4, (G) 50 mM and (H) 150 mM K-PO_4. For NaCl, no significant increase in the Aq value at the center of the sample could be observed when increasing the salt concentration. For KCl, the increase in salt concentration seemed to slightly increase the Aq value at the center of the sample. For Na-PO_4 and K-PO_4, the sample coverage was clearly increased, and the Aq value increased significantly when increasing the salt concentration.

Figure S10:

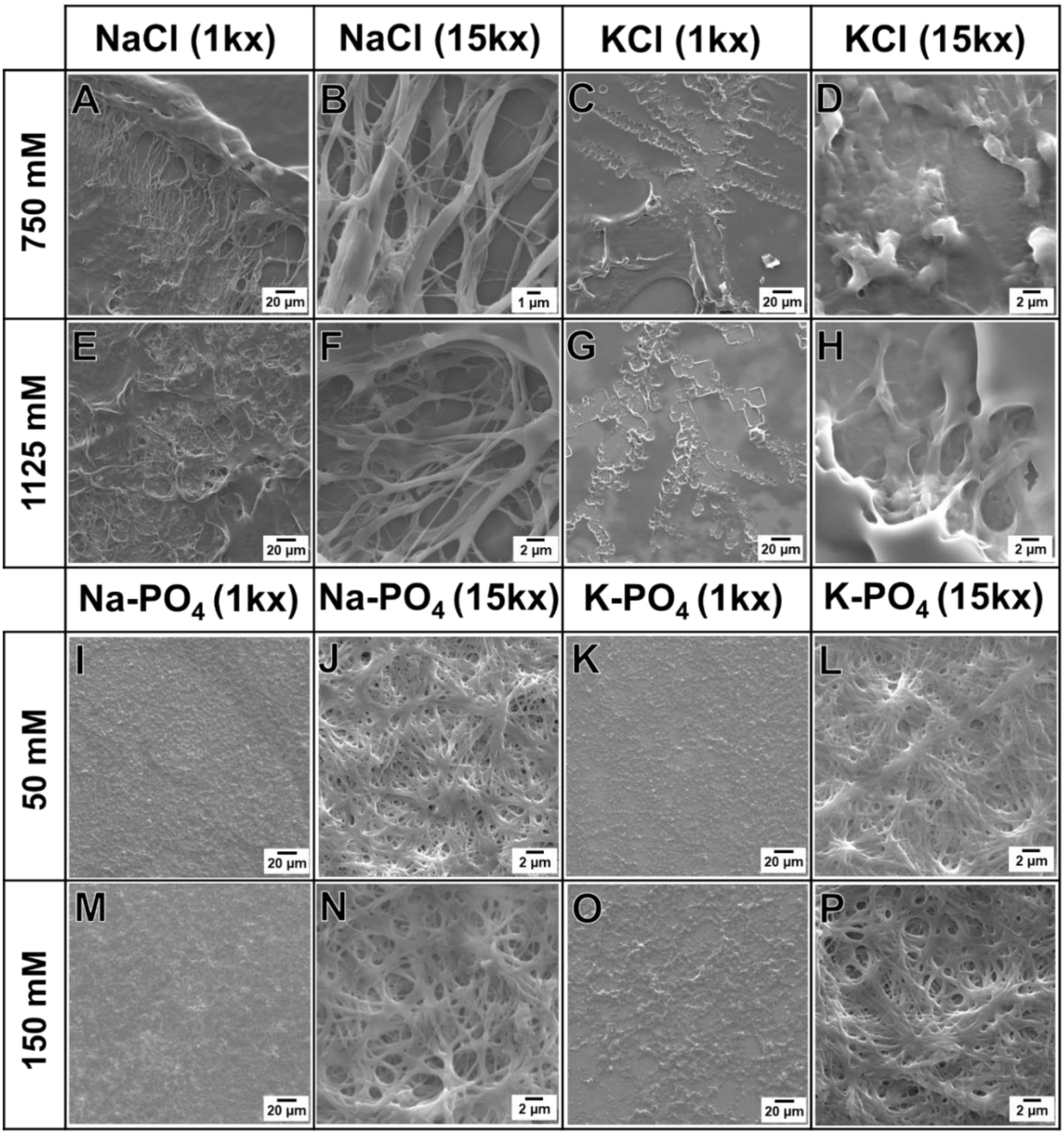

Figure S10: Effect of increased salt concentration on the surface roughness. SEM-based local detail images of fibrinogen samples after drying, crosslinking and washing mixtures of 2.5 mg/mL fibrinogen and different monovalent salts: (A, B) 750 mM NaCl, (C, D) 750 mM KCl, (E, F) 1125 mM NaCl, (G, H) 1125 mM KCl, (I, J) 50 mM Na-PO_4, (K, L) 50 mM K-PO_4, (M ,N) 150 mM Na-PO_4 and (O, P) 150 mM K-PO_4. All left images of the same salt show bigger regions of the samples at 1 kx magnification, while right side images show detailed parts of a zoomed area of the same region with 15 kx magnification. In general, the increase in salt concentration did not increase or suddenly trigger fibrinogen fiber formation. Similar structures/morphologies are seen for all samples prepared with the same salt, independently of the used salt concentration. 750 mM and 1125 mM NaCl yielded fibrous and planar regions comparable to the 375 mM NaCl sample presented in **Figure 5**. For KCl, no fiber formation was observed even by increasing the salt concentration, and the morphology of the fibrinogen layers was similar to the ones obtained for 375 mM KCl. Both concentrations of Na-PO_4 and K-PO_4 samples induced fiber formation with dense fiber networks.

Figure S11:

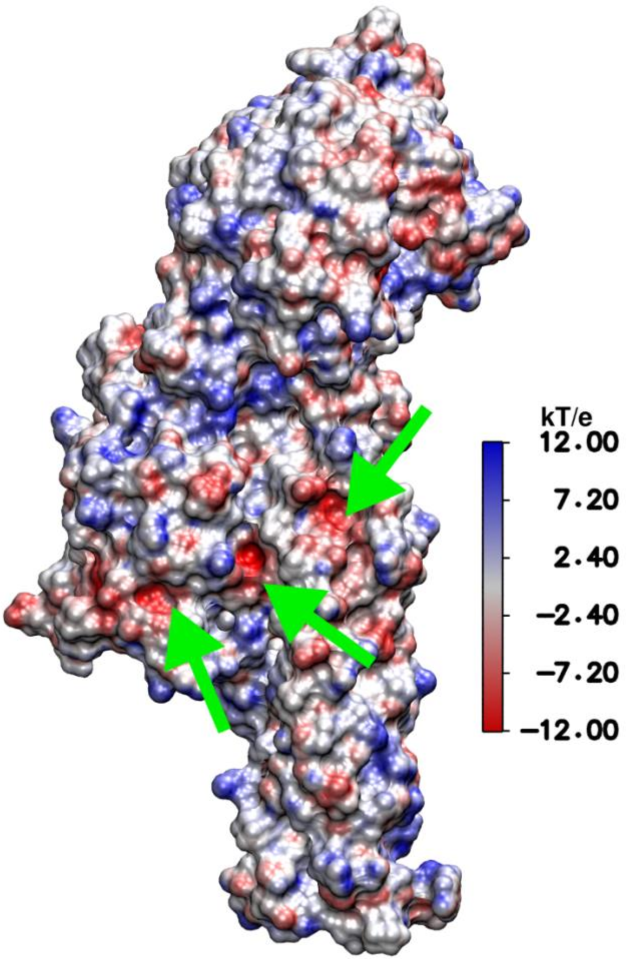

Figure S11: Electrostatic Potential coloring of Fg-D Domain calculated using APBS [2]. The location of the immobile Na^+ ions on the Fg-D domain are shown by arrows. The color scale represents the range of electrostatic potential values in units of kT/e at a temperature of 300 K.

Figure S12:

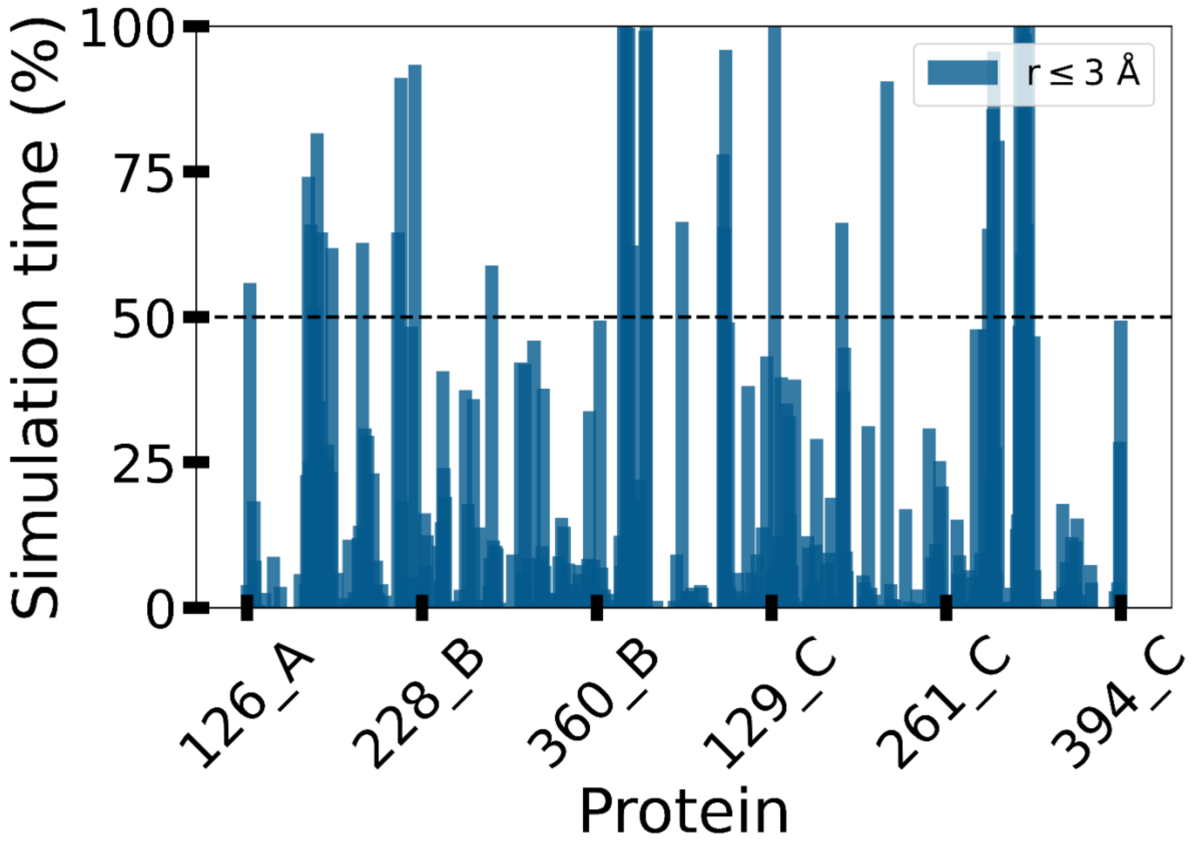

Figure S12: Ion retention within cutoff. Percentage of time for each protein residue sees an Na^+ within the specified cutoff distances. This gives us information about the protein residues that sees an ion for more than 50% of simulation time and based on this the position of mobile ions can be known.

Figure S13:

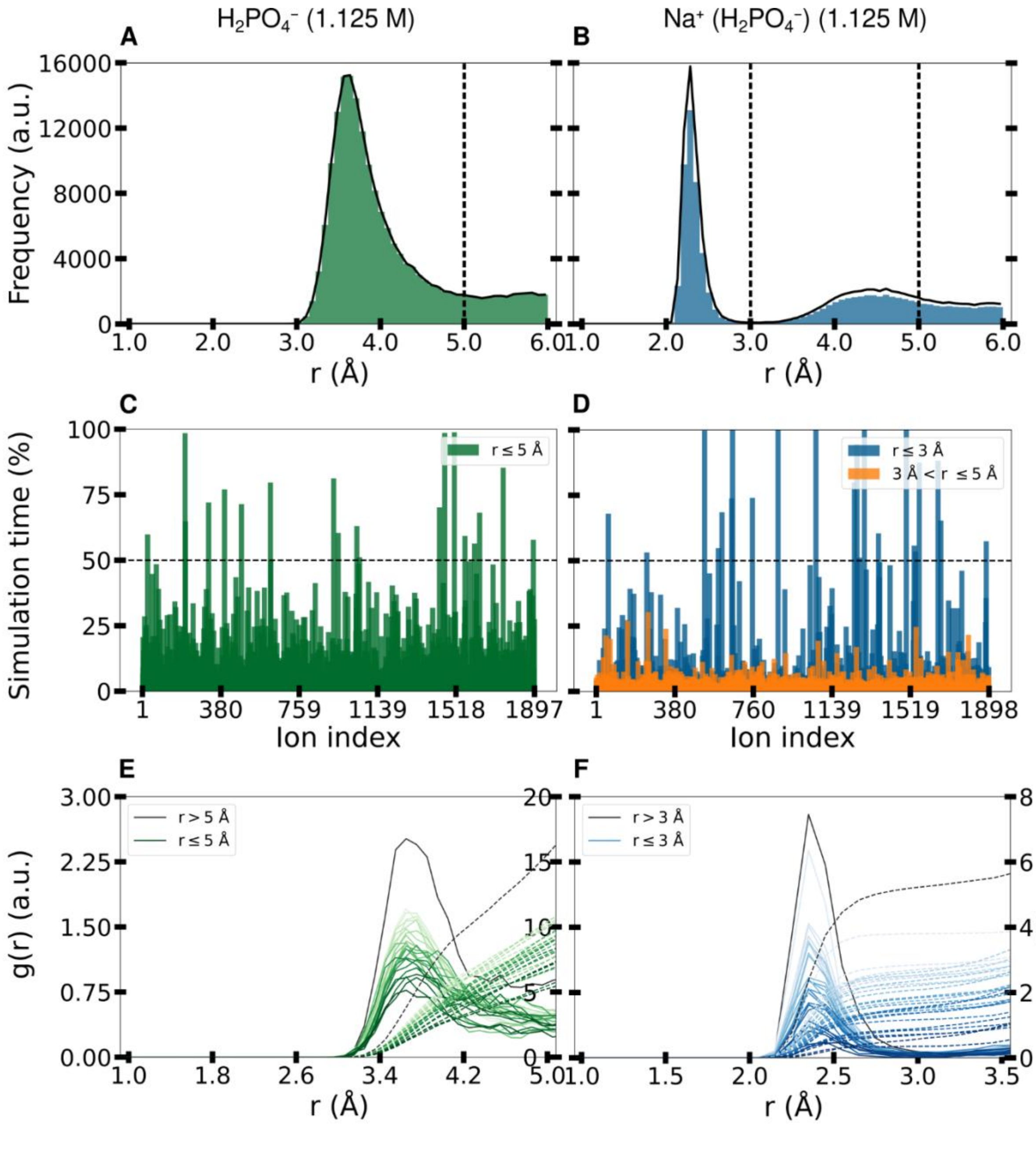

Figure S13: Simulation Analysis of Monovalent Phosphate Anion ($H_2PO_4^-$) and Its Influence on Cation Adsorption (Na^+ with $H_2PO_4^-$). (A, B) Distance distribution analysis: The distance is measured from the phosphorus atom of $H_2PO_4^-$ and Na^+} to all Fg-D protein atoms (excluding hydrogen atoms). A peak is observed between 3-5 Å for $H_2PO_4^-$, and two peaks are observed for Na^+ (between 2-3 Å and 3-5 Å). The black line indicates the location of the cutoff used for subsequent analysis. (C, D) Ion retention within cutoffs. Both the monovalent phosphate anion and Na^+ ($H_2PO_4^-$) show a greater percentage of time, with more than 50% of the simulation time spent close to the protein. (E, F) Hydration shell of immobile ions: This graph presents Radial Distribution Functions (g(r)) and Coordination Numbers (CN) of water oxygens around the immobile ions. g(r) is represented by a continuous line, and CN by a dashed line. The black curve represents the reference ion, which is not adsorbed to the protein during the simulation, while the colored curves represent the immobile ions. Both $H_2PO_4^-$ and Na^+ ($H_2PO_4^-$) exhibit disrupted hydration shells.

Figure S14:

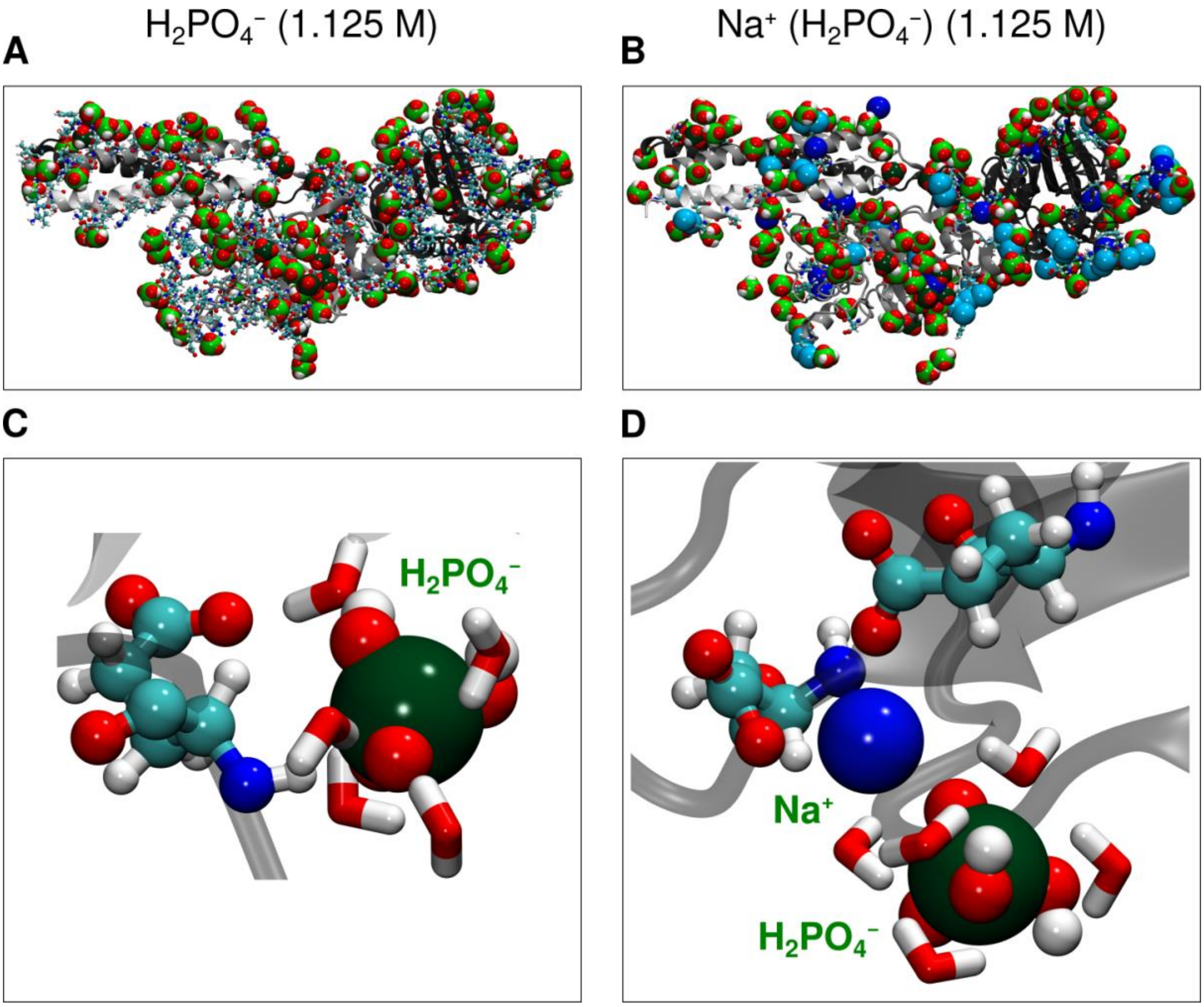

Figure S14: Representative snapshots of the mobile and immobile $H_2PO_4^-$ and Na^+ ions. (A, B) Both mobile ($H_2PO_4^-$: light green, Na^+: light blue) and immobile ions ($H_2PO_4^-$: dark green, Na^+: dark blue) that are in contact with the protein are shown. (C, D) Hydration shell around the immobile $H_2PO_4^-$ and Na^+ ions. The trapping of $H_2PO_4^-$ by the protein's atoms disrupts the water shell around the directly adsorbed ions. The trapping of Na^+ ion by the $H_2PO_4^-$ and the protein atoms is also seen.

Figure S15:

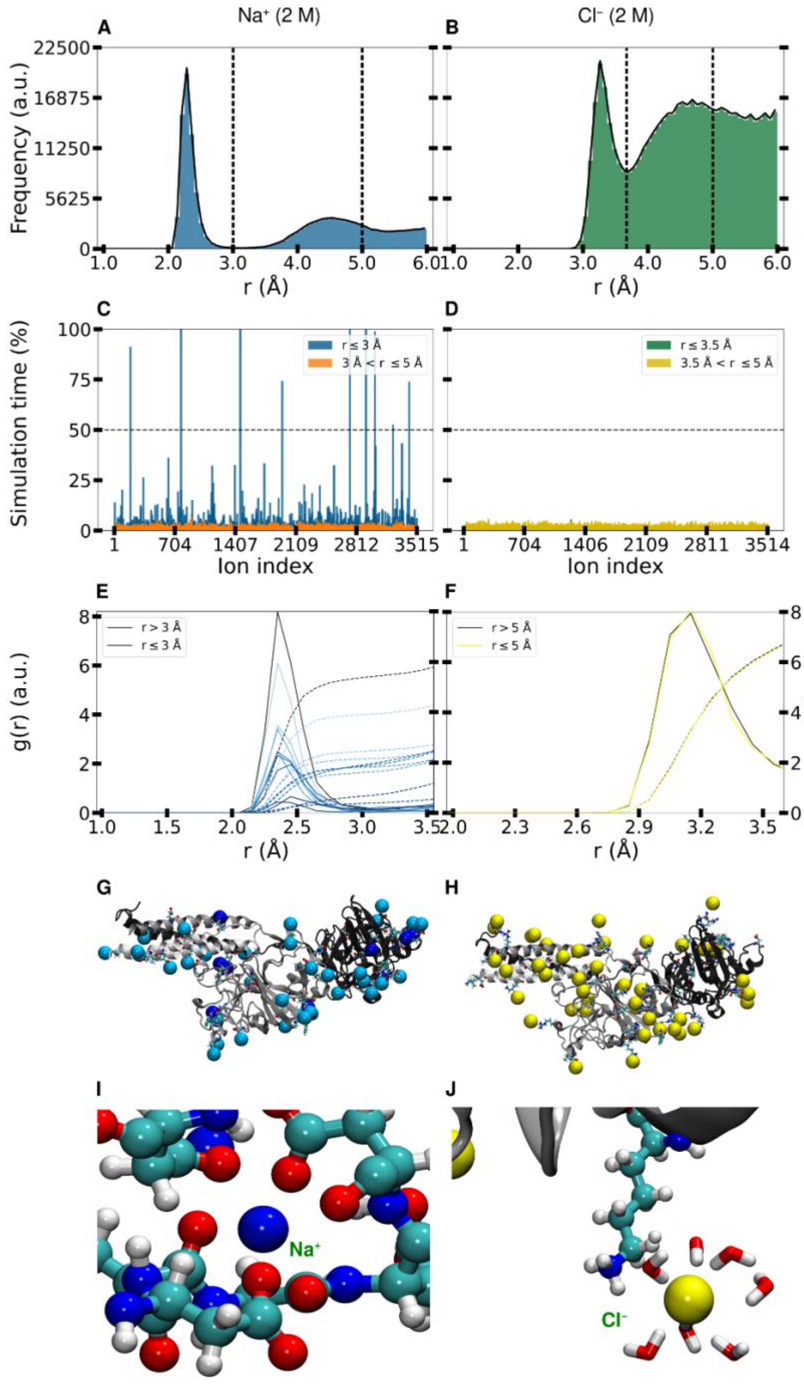

Figure S15: Simulation analysis and representative screenshots of Na^+ and Cl^- at 2 M concentration. (A, B) Distance distribution analysis of Na^+, Cl^- from the Fg-D protein atoms (excluding hydrogen atoms). Each graph displays two distinct peaks shown by the vertical dashed lines, with the minima of these peaks used as cutoffs for further analysis. (C, D) Ion retention within cutoff: Percentage of residence time for each ion within the specified cutoff distances from the Fg-D protein. The dashed horizontal line marks the 50% simulation time value. (C): Ion retention time for Na^+ ions in the first (blue) and second cutoffs (orange). (D): Ion retention time for Cl^- ions in the first (green) and second cutoffs(yellow). (E, F) Hydration shell of immobile ions. Radial distribution functions (g(r)) and coordination numbers (CN) of water oxygen atoms around the immobile ions. The g(r) is shown as a continuous line, while the CN is depicted with a dashed line. The reference state g(r) and CN of Na^+ and Cl^- are shown in black, immobile Na^+ in blue gradient and Cl^- in yellow. (G, H): Representative snapshots of the immobile and mobile cations. Dark blue corresponds to immobile Na^+, light blue to mobile Na^+ ions and yellow to mobile Cl^-. (I): Snapshot of Na^+ trapped by the negatively charged amino acids of protein with disrupted hydration shell. (J): Snapshot of Cl^- ion interacting with protein with full hydration shell.

References:

(1) Stamboroski, S.; Boateng, K.; Leite Cavalcanti, W.; Noeske, M.; Beber, V. C.; Thiel, K.; Grunwald, I.; Schiffels, P.; Dieckhoff, S.; Brüggemann, D. Effect of Interface-Active Proteins on the Salt Crystal Size in Waterborne Hybrid Materials. *Applied Adhesion Science* **2021**, *9* (1). https://doi.org/10.1186/s40563-021-00137-8.

(2) Jurrus, E.; Engel, D.; Star, K.; Monson, K.; Brandi, J.; Felberg, L. E.; Brookes, D. H.; Wilson, L.; Chen, J.; Liles, K.; Chun, M.; Li, P.; Gohara, D. W.; Dolinsky, T.; Konecny, R.; Koes, D. R.; Nielsen, J. E.; Head-Gordon, T.; Geng, W.; Krasny, R.; Wei, G.; Holst, M. J.; McCammon, J. A.; Baker, N. A. Improvements to the APBS Biomolecular Solvation Software Suite. *Protein Science* **2018**, *27* (1), 112–128. https://doi.org/10.1002/pro.3280.
